# Supplementary material for: ReNeuWell mental well-being app: protocol for a randomised controlled trial
Source: BMJ Open. 2025 Apr 12;15(4):e094557. doi: 10.1136/bmjopen-2024-094557 (PMC12094123; doi:10.1136/bmjopen-2024-094557)
Supplement: Supplementary data [file bmjopen-15-4-s001.pdf]

Supplementary Materials

for

The ReNeuWell mental wellbeing app:

Protocol for a randomised controlled trial

Luke A. Egan[1] and Justine M. Gatt[1,2,3*]

[1] Centre for Wellbeing, Resilience and Recovery, Neuroscience Research Australia, Randwick, NSW, Australia.

[2] School of Psychology, University of New South Wales, Kensington, NSW, Australia.

[3] Black Dog Institute, Randwick, NSW, Australia

***Corresponding author:** Associate Professor Justine Gatt; Phone: +612 9399 1812; Postal address: Neuroscience Research Australia, Barker St, Randwick, NSW 2031, Australia; E-mail address: j.gatt@unsw.edu.au

Egan LA, Gatt JM. *BMJ Open* 2025; 15:e094557. doi: 10.1136/bmjopen-2024-094557

Screening questions (in addition to K10)

1. How old are you?

2. Do you understand written English?

3. What country do you currently live in?
   - 🕊 Australia
   - 🕊 Canada
   - 🕊 Ireland
   - 🕊 New Zealand
   - 🕊 USA
   - 🕊 UK
   - 🕊 Other: ________________________

4. Do you currently suffer from a psychiatric or psychological condition? (e.g., anxiety disorder, depression, bipolar disorder, PTSD, phobia, psychosis, schizophrenia, eating disorder etc)

BMJ Publishing Group Limited (BMJ) disclaims all liability and responsibility arising from any reliance placed on this supplemental material which has been supplied by the author(s)

DEMOGRAPHICS

1	What is your age (in years)?	
2	Gender	• Male<br>• Female<br>• Non-binary / Other
3	What country do you currently live in?	Australia<br>Canada<br>Ireland<br>New Zealand<br>USA<br>UK
4	What state/ province/ county do you currently live in?	______________________________
5	What is your current marital status?	• Single<br>• Have a boyfriend/girlfriend<br>• Married / De facto (living with partner)<br>• Separated / Divorced<br>• Widowed
6	Height	_______ cm or _______ feet/inches
7	Weight	_______ kg or ________ pounds
8	Highest education completed	• High School<br>• Trade Certificate<br>• Graduate diploma or graduate certificate<br>• Undergraduate degree (Bachelor)<br>• Postgraduate degree (Master / PhD)
9	What is your current employment status?	• Full-time<br>• Part-time<br>• Studying and working<br>• Studying only<br>• Unemployed and looking for work<br>• Not doing paid work and not looking for work
10	What is your current main occupation?	______________________________
11	What is your role in your organization? (e.g., employee, manager, executive manager)	______________________________

12	Please think of the following 10-point scale like a ladder, representing where people stand in their society. People define society in different ways; please define it in whatever way is most meaningful to you.<br>At the top of the scale (“10”) are the people who have the highest standing in their society, and at the bottom (“1”) are the people who have the lowest standing in their society.<br>Where would you place yourself on this scale?	• 10. Highest standing in society<br>• 9.<br>• 8.<br>• 7.<br>• 6.<br>• 5.<br>• 4.<br>• 3.<br>• 2.<br>• 1. Lowest standing in society

HEALTH

1	Have you ever been diagnosed with a psychiatric or psychological condition? (e.g., eating disorder, anxiety disorder, depression, social phobia, PTSD)?	• Yes<br>• No<br>• Prefer not to answer
If “No” or “Prefer not to answer”, finish this section.		
2	Which psychiatric disorders have you been diagnosed with? Please tick all that apply.	• Eating Disorder<br>• Generalized Anxiety Disorder<br>• Panic Disorder<br>• Post-traumatic stress disorder (PTSD)<br>• Social Phobia / Social Anxiety Disorder<br>• Major Depression<br>• Bipolar Disorder (Manic Depression)<br>• Schizophrenia<br>• Obsessive Compulsive Disorder<br>• Personality Disorder (e.g. Borderline Personality Disorder)<br>• Tourette’s Disorder<br>• Other: ______________________________
3	What age were you when you were first diagnosed?	____________ years old
4	For how long did you suffer from this condition?	• Less than 1 year<br>• 1 – 3 years<br>• 3 – 5 years<br>• Ongoing

5	Did you receive treatment for this condition? Please tick all that apply.	• No treatment<br>• I received behavioural therapy (e.g., CBT): ___________ (please specify)<br>• I received medication treatment (e.g., antidepressants): ___________ (please specify)<br>• Alternative therapies (e.g., homeopathy, naturopathy): ___________ (please specify)

LIFESTYLE

1	On average, how many cups of caffeinated drinks (coffee, coke or energy drinks) do you drink per day?	• None<br>• 1 – 2 cups<br>• 3 – 4 cups<br>• Over 5 cups
2	Has there ever been a time when you smoked cigarettes regularly? That is, at least one cigarette per day for 3 months or longer?	🕊 Yes<br>🕊 No
If "No", go to question #9.		
3	How old were you when you first started smoking cigarettes regularly?	___________ years old
4	In total, how many years have you smoked cigarettes regularly?	___________ years
5	Over the time you smoked regularly, how many cigarettes on average did you smoke per day?	• 1-5 cigarettes per day<br>• 6-10 cigarettes per day<br>• 11-20 cigarettes per day<br>• 21-30 cigarettes per day<br>• More than a pack a day
6	Are you currently smoking cigarettes regularly?	🕊 Yes<br>🕊 No
If "Yes", go to question #7 then question #9.<br>If "No", go to question #8.		
7	Over the last year, how many cigarettes on average did you smoke per day?	• 1-5 cigarettes per day<br>• 6-10 cigarettes per day<br>• 11-20 cigarettes per day<br>• 21-30 cigarettes per day<br>• More than a pack a day

8	When did you stop smoking cigarettes regularly?	• I stopped smoking in the past 3 months<br>• I stopped smoking in the past 3-6 months<br>• I stopped smoking in the past 6-12 months<br>• I stopped smoking last year<br>• I stopped smoking when I was about: ____ years old
9	How often do you have a drink containing alcohol?	• Never<br>• Monthly or less<br>• 2-4 times a month<br>• 2-3 times a week<br>• 4 or more times a week
10	How many drinks containing alcohol do you have on a typical day when you are drinking?	• 1 or 2<br>• 3 or 4<br>• 5 or 6<br>• 7 to 9<br>• 10 or more
11	How often do you have 6 or more drinks on one occasion?	• Never<br>• Less than monthly<br>• Monthly<br>• Weekly<br>• Daily or almost daily
12	On average, how many times a week do you exercise (e.g., strength training, aerobic exercise, or activities such as running, swimming, strenuous walking or bike riding) for at least 30 minutes at a time?	• None<br>• 1 – 2 times<br>• 3 – 4 times<br>• 5 – 6 times<br>• Daily
13	On average, how many times a week do you eat fruit and vegetables?	• None<br>• 1 – 2 times<br>• 3 – 4 times<br>• 5 – 6 times<br>• Daily
14	How many hours of sleep do you usually get per night?	• Less than 3 hours<br>• 3 – 5 hours<br>• 6 – 8 hours<br>• Over 8 hours

APP SATISFACTION SURVEY

1. Overall, how important is it to you to improve your own wellbeing?

   • Very important
   • Moderately important
   • Neutral
   • Slightly important
   • Not important

2. How many apps have you previously downloaded or used that aim to improve your wellbeing (including meditation and mindfulness apps)?

   • None previously. This is my first time
   • 1-2 apps previously
   • 3 or more apps previously

3. Have you previously attended group classes like yoga and meditation?

   • No, never
   • Yes, I have attended a few classes previously
   • Yes, I attend yoga and/or meditative classes on a regular basis

4. Overall, how helpful do you feel the app was in increasing your mental wellbeing (e.g., mental health, positivity, emotional health)?

   • Very helpful
   • Quite a lot
   • Somewhat
   • A little
   • Not at all helpful

5. Overall, how helpful do you feel the app was in increasing your physical wellbeing (e.g., vigour, fitness, sleep)?

   • Very helpful
   • Quite a lot
   • Somewhat
   • A little
   • Not at all helpful

6. When using the app, how easy was it to find core features and navigate the app?

   • Very easy
   • Easy
   • Neutral
   • Difficult
   • Very difficult

7. Do you see the value in using the app?

- Yes, absolutely
- Yes, mostly
- Sometimes
- Not always
- Not at all

8. What is the likelihood you will continue using the app for your own wellbeing?

- Extremely likely
- Likely
- Neutral
- Unlikely
- Extremely unlikely

9. Do you prefer to use this app over other wellbeing apps?

- Yes
- No

If no, what other apps do you prefer and why? ______________________

10. What did you like the most about the app? ______________________

11. What activity did you like the most? ______________________

12. What did you dislike about the app? ______________________

13. What other information would you like to see in the app? ______________________

14. Other suggestions to improve the experience? ______________________

Adverse Event Assessment

Thank you for taking the time to share your answers and feedback. Your responses are anonymous and confidential, and the survey responses will be used to help develop our app further.

If any aspect of this survey or app caused you distress, please describe in the space below, including which items or features (otherwise you can leave it blank):

____
____
____
____

In the App Settings menu, we have provided contact details for Mental Health Support Services for your country, should you ever feel distressed and require additional mental health support.

PARTICIPANT INFORMATION STATEMENT

A randomised controlled trial of the ReNeuWell® wellbeing app

Chief Investigator: Dr Justine M. Gatt

1. What is this research study about?
The study measures people's responses to the ReNeuWell® app. You are invited to participate because you have installed the app on your phone.

2. Who is conducting this study?
The study is being run by Dr Justine M. Gatt from Neuroscience Research Australia (NeuRA) and the University of New South Wales (UNSW). The study is funded by the Mindgardens Neuroscience Network and the National Health and Medical Research Council (NHMRC).

3. Inclusion and exclusion criteria
Before you decide whether to participate, please know that the study is only recruiting people who:

1. are aged 18 years or older
2. can understand written English
3. have no current moderate to severe mental illness symptoms
4. are an iPhone user residing in Australia, New Zealand, UK, Ireland, USA, or Canada

4. Do I have to take part in this study?
Participation is voluntary. If you do not wish to participate, you can simply use the ReNeuWell® app as a regular user.

5. What does participation require, and are there any risks?
If you decide to participate, you will have 12 weeks of free access to one of two versions of the app. We wish to test which version of the app is more effective for increasing the user's wellbeing, and participants will be randomly assigned to one version or the other. For the first 6 weeks, the app will provide you with daily information and activities, and you will be instructed to use the app for at least 10 minutes a day, 6 days a week. You will take a short online survey before and after this 6-week period. For the final 6 weeks, you will be free to continue using the app as you see fit, and at the end of the 12-week period, you will take the third and final online survey. Each of the three surveys will take about 10 minutes to complete, and will be provided via the app.

The online surveys will ask about your mental health and wellbeing, lifestyle and work habits, and feelings about the app. If you experience any discomfort or distress while participating, you can stop at any time. The contact details of support services will be provided with each survey, and these details are also available below and on the ReNeuWell® NeuRA webpage: https://neura.edu.au/project/reneuwell

6. What are the possible benefits to participation?
Participants will have 12 weeks of free access to the ReNeuWell® app. Using the app may lead to increased mental wellbeing, and the results of this study will be used to develop future programs for improving people's wellbeing.

7. What will happen to the information I provide?
Your answers to the survey questions will be completely anonymous and confidential. The data will not contain any personally identifying information. In other words, there is no way for the researchers to know the participants' identities. If you decide to participate, the next screen will provide a button labelled "I agree to participate." By clicking this button, you can give your consent to participate.

8. How and when will I find out the results of the study?
The research team will publish the results in scientific journals and other academic outlets. No participant can be identified from these publications. The data will be stored for a minimum of 15 years after publication of the results. A summary of the results will also be available on the ReNeuWell® webpage: https://neura.edu.au/project/reneuwell

9. What if I want to withdraw from the study?
If you decide to participate but then change your mind, you can withdraw at any time, without penalty and without having to give a reason. If you would like to withdraw formally, you will have the option of clicking a button labelled "I wish to withdraw" at each occasion when you are prompted to continue with the study. You can also withdraw via the in-app options menu. After withdrawing, you can uninstall the app or continue using it as a paid user. If you withdraw, you will not be asked to complete any more surveys; however, we will not be able to withdraw your earlier survey answers, because the surveys are anonymous. If you withdraw, you will not be invited to participate in the study again.

10. What should I do if I have further questions about this study?
Please direct any questions about the study to the following research contact:

**Name**	Dr Luke Egan
**Position**	Research Assistant
**Telephone**	02 9399 1883
**Email**	l.egan@neura.edu.au

If you experience any feelings of distress at any stage during the study, or if you require additional support from someone not involved in the research, please access one of the following services (depending on your country of residence):

Australia

**Lifeline**	(telephone support 24 hours a day, 7 days a week)
Telephone:	13 11 14
Web:	https://www.lifeline.org.au/
**Beyond Blue**	(telephone support 24 hours a day, 7 days a week)
Telephone:	1300 224 636
Web:	https://www.beyondblue.org.au/get-support/get-immediate-support

New Zealand

**Lifeline Aotearoa**	(telephone support 24 hours a day, 7 days a week)
Telephone:	0800 54 33 54
Web:	https://www.lifeline.org.nz/

Egan LA, Gatt JM. *BMJ Open* 2025; 15:e094557. doi: 10.1136/bmjopen-2024-094557

UK

**Samaritans**	(telephone support 24 hours a day, 7 days a week)
Telephone:	116 123
Web:	https://www.samaritans.org/samaritans-ireland/

Ireland

**Samaritans**	(telephone support 24 hours a day, 7 days a week)
Telephone:	116 123
Web:	https://www.samaritans.org/samaritans-ireland/

USA

**National Suicide Prevention Lifeline**	(telephone support 24 hours a day, 7 days a week)
Telephone:	1800 273 8255
Web:	https://suicidepreventionlifeline.org/

Canada

**Canada Suicide Prevention Service**	(telephone support 24 hours a day, 7 days a week)
Telephone:	1833 456 4566
Web:	https://www.crisisservicescanada.ca/en/

If you have any complaints about any aspect of the study or how it's being run, please contact the UNSW Human Ethics Coordinator and cite the reference number: HC210302.

**Position**	Human Research Ethics Coordinator
**Telephone**	02 9385 6222
**Email**	humanethics@unsw.edu.au

If you have finished reading this Participant Information Statement, please go to the next screen to indicate whether you agree to participate.

Go to next screen →

CONSENT FORM

A randomised controlled trial of the ReNeuWell® wellbeing app

Declaration by the participant:

By clicking the "**I agree to participate**" button below, it will indicate that:

- I understand I am being asked to provide consent to participate in this research study.
- I have read the Participant Information Sheet.
- I provide my consent for the information collected about me to be used for the purpose of this study only.
- I understand that, if necessary, I can ask questions and the research team will respond to my questions.
- I freely agree to participate in the study as described and understand that I am free to withdraw at any time during the study without penalty and without having to give a reason.
- I understand that I can download a copy of the Participation Information Statement and Consent Form from the ReNeuWell® webpage: [insert URL here].

I agree to participate.

I decline to participate.

Egan LA, Gatt JM. *BMJ Open* 2025; 15:e094557. doi: 10.1136/bmjopen-2024-094557
