# Supplementary material for: ReNeuWell mental well-being app: protocol for a randomised controlled trial
Source: BMJ Open. 2025 Apr 12;15(4):e094557. doi: 10.1136/bmjopen-2024-094557 (PMC12094123; doi:10.1136/bmjopen-2024-094557)
Supplement: Author's manuscript [file bmjopen-2024-094557.draft_revisions.pdf]

BMJ Open is committed to open peer review. As part of this commitment we make the peer review history of every article we publish publicly available.

When an article is published we post the peer reviewers' comments and the authors' responses online. We also post the versions of the paper that were used during peer review. These are the versions that the peer review comments apply to.

The versions of the paper that follow are the versions that were submitted during the peer review process. They are not the versions of record or the final published versions. They should not be cited or distributed as the published version of this manuscript.

BMJ Open is an open access journal and the full, final, typeset and author-corrected version of record of the manuscript is available on our site with no access controls, subscription charges or pay-per-view fees (http://bmjopen.bmj.com).

If you have any questions on BMJ Open's open peer review process please email info.bmjopen@bmj.com

BMJ Open

The ReNeuWell mental wellbeing app: Protocol for a randomised controlled trial

Journal:	*BMJ Open*
Manuscript ID	bmjopen-2024-094557
Article Type:	Protocol
Date Submitted by the Author:	03-Oct-2024

Complete List of Authors:	Egan, Luke; Neuroscience Research Australia, Centre for Wellbeing, Resilience and Recovery<br>Gatt, Justine; Neuroscience Research Australia, Centre for Wellbeing, Resilience and Recovery; University of New South Wales - Kensington Campus, School of Psychology; Black Dog Institute

Keywords:	MENTAL HEALTH, Psychosocial Intervention, Adult psychiatry < PSYCHIATRY, Internet, Digital Technology, Mindfulness

SCHOLARONE™
Manuscripts

The ReNeuWell mental wellbeing app: Protocol for a randomised controlled trial

Luke A. Egan[1] and Justine M. Gatt[1,2,3*]

[1] Centre for Wellbeing, Resilience and Recovery, Neuroscience Research Australia, Randwick, NSW, Australia.

[2] School of Psychology, University of New South Wales, Kensington, NSW, Australia.

[3] Black Dog Institute, Randwick, NSW, Australia

***Correspondence:** Associate Professor Justine Gatt; Ph: +612 9399 1812; Postal address: Neuroscience Research Australia, Barker St, Randwick, NSW 2031, Australia; Email: j.gatt@unsw.edu.au

Word count:

Abstract: 401 words

Manuscript: 4698 words

Tables: 2 tables (193 words)

Figures: 1 figure

Supplementary Materials: 1 file (PDF document, 1469 words)

References: 4012 words

ORCID IDs

Luke A. Egan[1] ORCID ID: 0000-0003-0544-8851

Justine M. Gatt[1,2,3*] ORCID ID: 0000-0002-9276-6358

For peer review only - http://bmjopen.bmj.com/site/about/guidelines.xhtml

ABSTRACT

Introduction: The field of mental wellbeing interventions includes numerous studies of smartphone app-based programs, but there is a *research-to-retail gap* where many studies pertain to apps that are not publicly available, not used as standalone programs, or not tested in the general population, and many publicly available apps (or their proprietary in-app measures) have yet to be submitted to empirical testing. Furthermore, few wellbeing apps offer multi-component interventions, despite such interventions having demonstrated efficacy outside the smartphone context. In response to these openings in the literature and marketplace, we have developed ReNeuWell, an iPhone app designed to measure the user's mental wellbeing (via the validated COMPAS-W scale) and improve their wellbeing via a personalised, multi-component program of activities informed by the peer-reviewed evidence base. This paper describes the protocol for the pre-registered randomised controlled trial (RCT) of ReNeuWell, to test the app in adult participants from the general population of Apple App Store users. It is hypothesized that ReNeuWell users will experience significant increases in mental wellbeing and decreases in mental distress over the 6–12-week trial period, relative to users of an active control version of the app.

Methods and analysis: The RCT will recruit participants from Apple store users who choose to download ReNeuWell in the normal course of browsing the marketplace. Following consent, the app will randomly allocate participants to receive either the full version of the app or the active control version. Participants will be instructed to use the app for at least 10 minutes (at least one activity) per day for the first 6 weeks, and as they wish for the following 6 weeks. Trial outcomes will be measured via in-app surveys administered in weeks 1, 6, and 12. Data collection will begin when the app is officially launched on the Apple store. Data will be analysed using linear mixed models to estimate condition-by-time

For peer review only - http://bmjopen.bmj.com/site/about/guidelines.xhtml

interaction effects on the primary and secondary outcomes, and to assess whether any such effects are themselves moderated by other key variables.

Ethics and dissemination: This protocol has been approved by the Human Research Ethics Committee of the University of New South Wales (reference number: HC210302). Trial outcomes will be published in accordance with the pre-registered protocol described here, both in the peer-reviewed literature and on the registry website.

Registration: This protocol was pre-registered with the Australian New Zealand Clinical Trials Registry (registration approved on 4 August 2021; trial ID number: ACTRN12621001014842p).

For peer review only

Strengths and limitations of this study

- ReNeuWell is a newly developed iPhone app that addresses gaps in both the literature and the market. It is publicly available, incorporates a validated wellbeing measure (the COMPAS-W scale), and offers a personalised, multi-component program of evidence-based activities and resources.
- The RCT of ReNeuWell accounts for the placebo effect by offering an active control condition, and includes both a primary endpoint (6 weeks after baseline measures) and a secondary endpoint (12 weeks), allowing follow-up measurements of possible sustained benefits.
- The primary outcomes encompass both mental wellbeing and psychological distress (depression, anxiety, stress), which reflects the modern conception of mental health as comprising both minimal mental illness and optimal mental wellbeing.
- The participant screening process excludes those with moderate-to-severe distress, which may increase the likelihood of ceiling effects (for wellbeing outcomes) and floor effects (for distress outcomes).

For peer review only - http://bmjopen.bmj.com/site/about/guidelines.xhtml

- The study period encompasses a follow-up measure at 12 weeks after baseline, but if efficacy is demonstrated, this period could be expanded to longer time-frames to assess maintenance of effects.

For peer review only

For peer review only - http://bmjopen.bmj.com/site/about/guidelines.xhtml

Introduction

Optimal mental health is defined not only by minimal symptoms of mental illness but also by high levels of mental wellbeing (1-4). Low wellbeing, even without accompanying mental illness symptoms, can lead to poor psychosocial outcomes (1, 5), necessitating psychological programs that target wellbeing specifically, even in non-clinical populations (6, 7). Such interventions are increasingly offered via digital technologies such as websites and smartphone apps (8-10), some of which have been empirically tested (11, 12), but there remains the *research-to-retail gap*: much of the research pertains to apps that are not publicly available on app stores, have not been tested in the general population of app users, and/or are not offered as standalone programs (13, 14), which raises questions of ecological validity. Some studies even pertain to apps that were not given a product name, precluding other researchers from following up on the results. On the market side of the gap, it is common to find long-standing apps whose product descriptions make claims of improved mental health or wellbeing despite little-to-no empirical evidence (10, 12, 15-17), and apps that incorporate proprietary psychological measures (e.g., happiness questionnaires) that have not been validated in the peer-reviewed literature. Therefore, despite the large number of mental health apps that have been released over the years (10, 14, 15), there are still openings in the market for new, standalone apps that are both evidence-based and publicly available, as well as openings in the literature for peer-reviewed studies of such apps, incorporating established mental health measures.

The research-to-retail gap can be illustrated by considering the studies included in Linardon et al.'s recent meta-analysis of randomized controlled trials (RCTs) of app-supported mental health interventions (11). The meta-analysis incorporated 66 effects across 46 studies, finding that app-supported interventions can lead to small-to-moderate reductions in depression, anxiety, and stress; and small-to-moderate increases in mental wellbeing and

For peer review only - http://bmjopen.bmj.com/site/about/guidelines.xhtml

quality of life. However, few of the 46 papers described interventions that resembled the *typical experience* of downloading a publicly available app from an app store and using it as a *standalone* program without any accompanying resources or interactions (14). Across the 66 trials, only 14 publicly available mental health apps were examined, and of those apps, only Headspace was evaluated in more than one study. Of the three studies that examined Headspace, only one (18) involved a sample from the general population of adults to which the app is marketed. Only four other apps – Living with Heart (19), MoodKit, MoodMission, and MoodPrism – were studied in samples from the general population, and the latter three were evaluated in the same sample from a single study (20). Since Linardon et al.'s (11) meta-analysis, additional RCTs of mental wellbeing apps have been published (for example, (21-26)), and while it is beyond the scope of this paper to provide an exhaustive literature review, we have noted that few of these more recent trials were conducted in the general population of app store users (e.g., van Agteren et al. (26) sampled from the general adult population, although the participants were not recruited "naturally" through self-initiated downloads from an app store). Thus, there is actually little research on app-based wellbeing interventions within ecologically valid contexts. This is not a criticism of any individual study. For example, it is perfectly worthwhile to test whether a given mental health app benefits members of a sub-population (e.g., people with diagnosed major depressive disorder); we are simply noting that such studies are less relevant to our understanding of how such apps affect users in general, to whom all the top mental health apps are marketed (14).

For a new wellbeing app to be potentially beneficial, it should offer something beyond the contents of existing apps, while being grounded in an established model of mental wellbeing. A recent narrative review (14) examined the top 20 mental health apps on the Apple and Google stores, which together accounted for 98% of monthly active users (MAUs,

For peer review only - http://bmjopen.bmj.com/site/about/guidelines.xhtml

i.e., unique individuals who used a mental health app at least once in the one-month reference period). Of these apps, 18 provided unguided, standalone programs, most of which fell into one of three categories: mindfulness/meditation, journaling/self-monitoring, or conversing with an artificial intelligence (AI) chatbot. The top two mindfulness/meditation apps – Headspace and Calm – together accounted for approximately 70% of MAUs. Clearly, those seeking mental health support via smartphone apps either prefer mindfulness/meditation exercises or have few alternatives. The latter is plausible: Lagan et al.'s recent review of 278 mental health apps (15) found that the three most common activities (provided by hundreds of different apps) were self-monitoring, journaling, and mindfulness exercises. In contrast, exercises from evidence-based therapies, such as cognitive behavioural therapy (CBT) or acceptance and commitment therapy (ACT), were offered by far fewer apps (<60 for CBT and <20 for ACT). Thus, there may be unmet demand for new apps that provide activities and resources not included in many (if any) of the current offerings. The positive psychology literature describes numerous exercises and strategies to raise mental wellbeing whose efficacy has been demonstrated in non-app interventions (6, 7, 27); these are prime candidates for conversion to app-based formats where possible. Lagan et al.'s (15) review also highlighted "the lack of comprehensive apps that would facilitate multiple diverse uses" (p. 8). Although a user could download multiple apps to cover a range of activities, there are numerous drawbacks of this approach (17), including the obvious inconvenience, the possibility of receiving contradictory notifications or advice from two or more apps, and (with paid apps) the expense.

Furthermore, van Agteren et al.'s recent meta-analysis of mental wellbeing interventions (7) concluded that the two kinds of intervention most consistently associated with positive outcomes were mindfulness-based activities and *multi-component* positive psychology interventions (comprising two or more positive psychology techniques in a single

For peer review only - http://bmjopen.bmj.com/site/about/guidelines.xhtml

program). Although this meta-analysis was not limited to app-based or digital interventions, it clearly suggests that multiple activities should be considered in programs to improve mental wellbeing. Despite this, a recent review of 19 popular mental health apps (28) found that 8 of the apps offered only a single evidence-based treatment component (e.g., meditation only), with an average of 2.32 components per app across all 19 apps, none of which offered more than 5 components (for comparison, van Agteren et al.'s meta-analysis covered over 15 types of positive psychology intervention). Moreover, Marshall et al. (29) have observed that many mental health apps "only offer singular or novelty interventions that do not qualify as comprehensive therapeutic treatments and/or diagnostic instruments" (p. 217).

Given the preceding considerations, we have developed the forthcoming iPhone app "ReNeuWell" (a portmanteau of "Resilience, Neuroscience, Wellbeing"), which may address the opening for a standalone, publicly available, multi-component wellbeing app for the general adult population. ReNeuWell comprises psychoeducational materials, positive psychology exercises, and numerous other activities and resources, with the aim of increasing mental wellbeing in users. We included as many evidence-based activities and resources as possible, within the inherent limitations of app-based delivery as well as budgetary and logistical constraints. To track each user's wellbeing and provide them with a personalised program, ReNeuWell incorporates a comprehensive measure of mental wellbeing – the COMPAS-W scale (5) – which has been validated in numerous prior studies with regard to both its psychometric properties and various correlates including health behaviours, work productivity, genetics, neural activity, and cognitive functioning (3, 5, 30-35). Indeed, the entire app is grounded in the COMPAS-W model of wellbeing. Each activity is designed to target one of the six dimensions of wellbeing measured by the COMPAS-W, and the psychoeducational materials educate the user on the six dimensions and how each contributes to total wellbeing. ReNeuWell was invented by the present authors alone, with the software

For peer review only - http://bmjopen.bmj.com/site/about/guidelines.xhtml

programming and graphic design implemented by the digital development studio Miroma Project Factory. Here we describe the protocol for a pre-registered RCT of ReNeuWell. We hypothesize that the app will produce significant improvements in mental wellbeing and reductions in psychological distress, relative to an active control version of the app.

Method

Study design

This protocol is for a single-blinded, active-controlled RCT with two conditions (training versus active control) and three measurement occasions (surveys in week 1, week 6, and week 12). The 6- and 12-week endpoints were selected because we have previously found significant effects from a smaller set of similar activities after 6 weeks (27).

Materials and procedure

The ReNeuWell app

ReNeuWell will be commercially available on the Apple store to the general public. After downloading the app and opening it for the first time, users will receive the option to continue as an ordinary paying user or to enrol in the RCT and receive free access for the 12-week study period. Thus, everyone who downloads ReNeuWell will be invited to participate. Those who select the paid version will proceed to the full version of the app and will not be included as participants. Those who select the RCT option will proceed to the participant information statement and consent form, followed by screening questions to confirm eligibility. If they provide consent and pass the screening questions, participants will be randomised automatically by the app (via alternating allocation with a 1:1 ratio) to either the training condition (providing the full version of the app) or the active control condition (providing a dummy version of the app). Following randomisation, users will create an

For peer review only - http://bmjopen.bmj.com/site/about/guidelines.xhtml

For peer review only

account and proceed to the app proper.

ReNeuWell comprises two main components: a recurring survey and a suite of activities. The survey constitutes the COMPAS-W scale (5), which measures the user's wellbeing along six dimensions: Composure (dealing effectively with stress or adversity), Own-worth (a sense of autonomy and self-respect), Mastery (self-confidence, perceived control over one's environment), Positivity (happiness, optimism), Achievement (setting and pursuing goals), and Satisfaction (with one's life, health, and relationships). Upon first using the app, and every six weeks thereafter, participants complete the COMPAS-W, and their scores on the six dimensions are used by the app to generate a personalised program of daily activities for the next six weeks. Users are not shown their exact numeric scores on the COMPAS-W, but rather a screen titled *Your Results*, which provides both visual and written feedback. The written feedback comes in one of three forms, corresponding to the three categories of mental health described by Keyes (1, 2) and established for the COMPAS-W (5): *languishing, moderate wellbeing*, and *flourishing*. Participants in the RCT will also complete additional measures within the recurring surveys. However, those in the active control condition will not be shown the *Your Results* screen.

After viewing their COMPAS-W results, participants in the training condition will be informed that the app will now generate their personalized program of activities, which constitutes the second main component of ReNeuWell. The app currently offers 18 activities, some of which come in multiple versions to cover different aspects of the relevant topic. Depending on the study outcomes, future updates to the app may add new activities or variations of existing ones. Table 1 lists each activity, along with citations of peer-reviewed studies that informed its development or support its inclusion in the app. In contrast, the active control participants will not be told that they will receive a personalised program, because the reduced app contains only a single program with three activities, designed to be

For peer review only - http://bmjopen.bmj.com/site/about/guidelines.xhtml

neutral with regard to mental wellbeing (i.e., harmless but not beneficial). These three activities are named Acts of Novelty, Life Reminiscence, and Self Esteem, and are based on the active control conditions implemented and tested in a RCT of an online wellbeing intervention (27).

Once a participant has received their program, every time they open the app (for the next six weeks), they will be taken to the *dashboard* (the "hub" screen of the app), from which they can browse their schedule of activities and select one or more to complete each day. By default, the app generates a reminder notification for users who have not opened the app for more than 72 hours, reminding them to use the app and suggesting that they try another activity. The dashboard also provides access to additional resources and features, including psychoeducational materials on mental wellbeing (with the active control app containing fewer psychoeducational resources). All users can also access contact information for mental health support services (e.g., 24-hour crisis hotlines), report an issue (e.g., a software glitch), or withdraw from the RCT via the dashboard. Participants can withdraw at any time without penalty and without having to provide a reason. Those who withdraw will have the option of continuing as a paying user.

INSERT TABLE 1 ABOUT HERE

Instructions and timeline

Participants in both conditions will be instructed to use ReNeuWell for at least 10 minutes per day for the first six weeks of the 12-week study period. After week 6, participants are instructed to use the app as they see fit and informed that they will receive their final survey in week 12. For this reason, week 6 is the primary endpoint of the study, with week 12 as the follow-up.

For peer review only - http://bmjopen.bmj.com/site/about/guidelines.xhtml

In both conditions, each participant's schedule of daily activities is organised into weeks, with participants able to see the current week of their six-week program and any previous weeks if applicable. Participants can navigate to earlier weeks if they wish to complete an activity that they skipped, but they cannot access future activities ahead of schedule. In order for the next week of the program to be unlocked, the user must open the app at least once during the current week. Therefore, although our RCT is designed as a 12-week study, it will be possible for participants to take longer than 12 weeks to complete their programs. However, the app automatically records when a given activity or survey has been completed, allowing us to control statistically for any divergences from the intended 12-week schedule. After completing the week 12 survey and activities, training participants will be offered an additional four weeks of free access to the app (the full version this time) while control participants will be offered 12 weeks of free access to the paid version. Thus, control participants will not know that they received a reduced version of the app until they unlock the full version after week 12. Indeed, the Participant Information Statement informs prospective participants that they will be randomly allocated to one of two versions of the app, but they are not told that one of the versions is a reduced version and will not be told which version they received. Each participant's app simply appears to them under the name "ReNeuWell." The study timeline is illustrated in Figure 1.

INSERT FIGURE 1 ABOUT HERE

Participants

Recruitment

The participants will be recruited from the general population of iPhone users who choose to download ReNeuWell from the Apple store. The RCT will not happen within a

For peer review only

For peer review only - http://bmjopen.bmj.com/site/about/guidelines.xhtml

fixed calendar period; rather, prospective participants will be invited to participate in an ongoing manner, whenever they first open the app. All aspects of each condition (such as instructions, activities, and educational materials) will be delivered purely via the app. Therefore, participants will not need to interact with another app, a website, or any other entity (including the researchers) at any stage of the study.

Sample size

The minimum sample size was calculated with the software package G*Power (ver. 3.1.9.2) (36, 37). The type-1 error rate was set at the conventional level of 0.05. With the desired power of 95% and assuming a correlation of 0.5 between the repeated outcome measures, our calculations determined that a total sample size of 324 participants would be needed to detect a small group-by-time interaction effect (partial $\eta^2 = 0.01$) with two conditions and two measurement occasions (our study has three measurement occasions, but the second occasion is the primary endpoint of the study, and a power calculation with two occasions is stricter than one with three). However, given that app-based interventions demand relatively low commitment from participants, it is reasonable to assume an attrition rate of at least 50% by the second measurement occasion. Indeed, a recent meta-analysis of RCTs of smartphone interventions (38) found attrition rates of 53.4% between weeks 9 and 12. Therefore, we set our target sample size at 486 participants, with 243 in each condition.

Inclusion criteria

Prospective participants will be informed that participation in the RCT is open to those who are willing to use the app for at least 10 minutes per day for at least the first 6 weeks of the 12-week study period (and up to 12 weeks), and willing to complete a 10-minute survey in weeks 1, 6, and 12. Participants will also need to meet these additional eligibility criteria:

For peer review only - http://bmjopen.bmj.com/site/about/guidelines.xhtml

- Aged 18 years or older (for informed consent),
- Able to understand written English (ReNeuWell is currently available in English only),
- Absence of moderate-to-severe symptoms of mental illness, as measured by a single-item question and scores on the K10 screening questionnaire (39),
- Residing in one of the following countries: Australia, New Zealand, United Kingdom, Ireland, United States, Canada (English-speaking countries),
- Possesses an iPhone (or iPod Touch) with operating system iOS 14.0 or later (although we intend to release the app on Android devices in the future, pending funding).

When ReNeuWell is first released on the Apple store, participation in the RCT will be an option only for users residing in Australia. We have approval to recruit participants in all six of the aforementioned countries, but we have decided to confine the study to Australia initially, so that we will be well-positioned (being within our home country) to address any unexpected issues with the app or the trial. Barring any major technical issues or adverse events in this initial period, participation will be extended to the other five countries (and further countries pending future developments).

Screening questions

The screening questions ask for the user's age, whether they understand written English, which country they reside in, and whether (yes/no) they currently suffer from a psychological or psychiatric condition. In addition, the screening questions include the Kessler Psychological Distress Scale (K10) (39). K10 scores of 25 or higher are considered indicative of moderate-to-severe distress and therefore of the respondent being ineligible for study participation. Those who do not pass the screening questions will be redirected to a

For peer review only - http://bmjopen.bmj.com/site/about/guidelines.xhtml

screen whereby they can continue using the app as a paying user, if they wish. The screening questions (except the K10) are provided in the Supplementary Materials.

Survey measures

At each measurement occasion (i.e., when the participant first opens the app in week 1, week 6, and week 12), the participants will complete a 10-minute survey and will not be able to access further activities until the survey is complete. Each survey comprises a range of questionnaires, but not all measures will be administered at each measurement occasion. Table 2 outlines the questionnaire timings.

INSERT TABLE 2 ABOUT HERE

Kessler Psychological Distress Scale (K10)

The Kessler Psychological Distress Scale (K10) (39) will be administered during the initial screening process (as already described) and also at the beginning of the second and third surveys. A moderate-to-high K10 score at the screening stage will make the user ineligible for further participation, with K10 scores again assessed at the second and third survey time-points. The K10 comprises 10 questions on various symptoms of psychological distress (e.g., depression and anxiety symptoms), with a 5-point response scale ranging from *none of the time* to *all of the time*. Respondents are instructed to answer the questions in terms of how they have been feeling *over the past 30 days*. The K10 has exhibited acceptable reliability and validity in previous studies, and is one of the most frequently used measures in mental health research and practice (40-42).

Demographics, Health, and Lifestyle questionnaires

For peer review only - http://bmjopen.bmj.com/site/about/guidelines.xhtml

The Demographics questionnaire will ask about characteristics including marital status, highest education, and current occupation. The Health questionnaire will ask about the user's psychiatric history, including whether they have been diagnosed with and treated for a mental illness in the past. The Lifestyle questionnaire will ask about the user's consumption of caffeine, cigarettes, and alcohol, as well as their diet, exercise, and sleep habits. The Demographics, Health, and Lifestyle questionnaires are provided in the Supplementary Materials.

COMPAS-W Scale

The COMPAS-W Scale (5) is a 26-item measure of overall mental wellbeing and the six subcomponents of Composure, Own-worth, Mastery, Positivity, Achievement, and Satisfaction. Each item is a statement with a 5-point response scale ranging from *strongly disagree* to *strongly agree*. The respondents are instructed to answer each statement in terms of how they feel *most of the time* for the week 1 survey, and then how they felt *over the past month* for later time-points. The COMPAS-W Scale has exhibited acceptable reliability and validity in several previous studies (5, 30-33, 43).

Depression, Anxiety, and Stress Scales – 21-Item Version

The Depression, Anxiety, and Stress Scales – 21-item version (DASS-21) (44, 45) is a measure of psychological distress (i.e., symptoms of depression, anxiety, and stress) comprising 21 statements with a 4-point response scale ranging from *did not apply to me at all* to *applied to me very much or most of the time*. The respondents are instructed to answer each statement in terms of how much it has applied to them *over the past week*. The DASS-21 has exhibited acceptable reliability and validity in prior non-clinical research (46, 47).

For peer review only - http://bmjopen.bmj.com/site/about/guidelines.xhtml

Health and Work Performance Questionnaire Scales (Employee Version)

Two sections from the employee version of the World Health Organization Health and Work Performance Questionnaire (48, 49) – scale B9 and scale B12 – will be used to measure participants' work performance. Scale B9 comprises five items asking how many days in the past four weeks the respondent missed an entire workday or part of a workday (for health or other reasons), or performed work outside their normal working hours. Scale B12 contains seven items assessing the respondent's appraisal of their own work performance in the past 4 weeks, with a 5-point response scale ranging from *none of the time* to *all of the time*.

App Satisfaction Survey

In the week 6 survey, participants will receive the ReNeuWell App Satisfaction Survey, containing a range of questions regarding the participant's opinion of the app and what they liked or disliked about it. This questionnaire is provided in the Supplementary Materials.

Adverse Event Assessment

Each survey will conclude with an Adverse Event Assessment, constituting a text box in which the participant can report any aspect of the app or survey that caused discomfort or distress. If the participant has no adverse events to report, they will leave the box empty and finish the survey. The Adverse Event Assessment is provided in the Supplementary Materials.

App usage metrics

Data regarding participant usage metrics (e.g., which activities they completed, how much time they spent using the app) will be recorded within the app and via Google back-end

For peer review only - http://bmjopen.bmj.com/site/about/guidelines.xhtml

software (Firebase).

Anonymity and confidentiality

All data collected by ReNeuWell will be automatically uploaded to a secure, dedicated server (located in Australia). On this server, users' account data (i.e., names and email addresses) are stored separately from their survey data and are only accessible using a permissions-based system. This ensures security of the data over and above encryption. The survey data will not include any personally identifying information but rather a trial participant ID number that is not shared with the user. Only the research team and app development team (Miroma Project Factory) will have access to the server. The deidentified data will then be downloaded directly from this server onto the secure internal server at Neuroscience Research Australia (NeuRA) for storage and analysis. Only the research team will have access to the data.

Data availability

Per ethical requirements and participant consent, data will not be shared beyond the research team.

Statistical analysis

The primary outcomes for this RCT will be the participants' levels of both mental wellbeing (as measured by the COMPAS-W) and psychological distress (as measured by the DASS-21). The secondary outcomes will be psychological distress as measured by the K10 and work performance. Potential moderator variables include levels of usage and enjoyment of the app, past mental illness, lifestyle habits, baseline mental health levels, and demographic characteristics such as age, sex, education, or occupation.

For peer review only - http://bmjopen.bmj.com/site/about/guidelines.xhtml

The initial analyses will be conducted on an intention-to-treat basis, and followed by per-protocol analyses. Missing values will be estimated via multiple imputation. The hypotheses will be tested via a linear mixed model estimating the group-by-time interaction effects on the outcome variables. It is hypothesized that levels of mental wellbeing (COMPAS-W) will increase and levels of psychological distress (DASS-21) will decrease in the training condition relative to the control condition. We will also test for group-by-time interaction effects on work performance and the K10 measure of psychological distress. Any interaction effect may itself be moderated by variables such as age, sex, education, occupation, psychiatric history, lifestyle habits, app usage metrics, or baseline mental health levels.

Ethics and dissemination

This protocol has been approved by the Human Research Ethics Committee of the University of New South Wales (reference number: HC210302) and pre-registered with the Australian New Zealand Clinical Trials Registry (registration approved on 4 August 2021; trial ID number: ACTRN12621001014842p). Our findings will be published in accordance with this protocol, both in the peer-reviewed literature and on the registry website.

Public involvement

Because ReNeuWell is designed for use by the general adult population, it was appropriate to conduct several rounds of consultations with members of the general public throughout the app's development. During initial design stages, we conducted user acceptance testing (UAT) with volunteers from the general public, who provided feedback on the app's design, colours, contents, and features. The app was subsequently refined over numerous iterations, after which beta testing was conducted with local community members

For peer review only - http://bmjopen.bmj.com/site/about/guidelines.xhtml

for feedback on usability and acceptability of the app, including identification of any bugs or feature issues. The app was then refined further and retested in-house across multiple iterations, before being “soft launched” on the Apple store for additional testing by a new group of local community members (i.e., volunteers could download ReNeuWell from the Apple store for testing purposes).

Discussion

The field of app-based interventions to improve mental wellbeing has yielded promising findings to date, but there remains a *research-to-retail gap* (13, 14), where many studies pertain to apps that are not publicly available, have not been tested in the general population, and/or contain proprietary wellbeing measures that have not been validated in the peer-reviewed literature. Furthermore, few previous studies have been conducted in an ecologically valid setting, where a user freely chooses to download a publicly available app from a mainstream app store and then uses the app as a standalone program without ever needing to interact with another person or entity. On the market side of the gap, many popular apps have remained available on mainstream app stores for years without ever being submitted to peer-reviewed empirical assessment, and some of these apps have product descriptions that make claims of efficacy despite the lack of empirical evidence (10, 12, 15, 29).

Alongside the research-to-retail gap, another limitation is that most wellbeing apps cover only one or a few techniques (28, 29). The literature suggests that mindfulness exercises, which are among the techniques most commonly offered by wellbeing apps (14, 15), can indeed be effective for increasing wellbeing (7), but multi-component interventions (comprising a range of positive psychology exercises) also have efficacy (7) but have not been offered by many apps previously (28). Furthermore, although some wellbeing apps offer

For peer review only - http://bmjopen.bmj.com/site/about/guidelines.xhtml

evidence-based exercises (28), few if any are informed by a comprehensive, empirically validated model of wellbeing. The COMPAS-W framework (5) provides not only a multi-dimensional description of total wellbeing, but also a validated scale to measure each dimension and overall wellbeing.

ReNeuWell is a newly developed iPhone app, grounded in the COMPAS-W model of mental wellbeing (5), which addresses these openings in the literature and in the marketplace, and its evaluation in our upcoming RCT will hopefully address some of the key gaps in our knowledge of mental wellbeing apps and their effectiveness. ReNeuWell provides a tailored program of daily activities and other resources designed to increase wellbeing in one or more of the six COMPAS-W dimensions. We aim to determine whether this publicly available, standalone app can promote greater wellbeing and reduce distress in the general adult population of Apple store customers who choose to use it.

For peer review only

For peer review only - http://bmjopen.bmj.com/site/about/guidelines.xhtml

Acknowledgements

The app was developed by Miroma Project Factory in consultation with the authors.

Funding statement

Costs to build the app and run this project were funded by a Mindgardens Neuroscience Grant awarded to JMG. LAE was supported by a Mindgardens Neuroscience Grant awarded to JMG, and a National Health and Medical Research Council (NHMRC) Project Grant (1122816) awarded to JMG. JMG was supported by a National Health and Medical Research Council (NHMRC) Project Grant (1122816). The study funders had no role in designing the present protocol, nor will they have any role in data collection, management, analysis, interpretation, or dissemination.

Conflicts of interest

LAE, JMG, NeuRA and UNSW NSi will receive proportional payments from any net income received from paying users of the ReNeuWell app. The trial has been pre-registered with ANZCTR and all trial outcomes will be reported in accordance with the published protocol. All trial outcomes will be made publicly available, including null results, which will be used to improve the app for future use and potential re-trials, pending funding availability.

Author contributions

LAE conducted the literature review, drafted the initial paper and subsequent versions, and contributed to the original copy of the app. JMG conceptualised the original ReNeuWell app and its design (including copy content), formulated the clinical trial study design and aims of the paper, and helped edit the original draft and revised versions. Both authors revised and edited the final version of the paper.

For peer review only - http://bmjopen.bmj.com/site/about/guidelines.xhtml

Table 1. List of activities offered in the full version of ReNeuWell, along with citations of peer-reviewed studies that informed their development or support their inclusion in the app.

**Title/Topic**	**Citations**
Acts of kindness	(27, 50-53)
Assertiveness training	(54-58)
Body language	(59-62)
Building strengths	(6, 63-65)
Colouring mandalas	(66-69)
Controlled breathing	(70-74)
Facial expressions	(75-78)
Gratitude diary	(6, 63, 79-83)
Guided meditation	(72, 84-89)
Mindfulness	(84, 87, 90-96)
Personal values	(97-100)
Positive event scheduling	(101-107)
Positive reminiscence	(27, 108-112)
Recent achievements	(113-117)
Self-compassion	(27, 118-123)
Setting goals	(124-130)
Showing empathy	(51, 131-135)
Stress and coping	(136-140)

For peer review only - http://bmjopen.bmj.com/site/about/guidelines.xhtml

Table 2. Questionnaires delivered at each time-point.

	Screening (Start of week 1)	Survey 1 (Start of week 1)	Survey 2 (Start of week 6)	Survey 3 (Start of week 12)
Screening questions	✓			
K10[a]	✓		✓	✓
Demographics		✓		
Health		✓		
Lifestyle		✓		
COMPAS-W[b]		✓	✓	✓
DASS-21[c]		✓	✓	✓
HPQ[d]		✓	✓	✓
App satisfaction			✓	
Adverse event assessment		✓	✓	✓

Notes: [a]K10: Kessler Psychological Distress Scale – 10-item version. [b]COMPAS-W: Composure, Own-worth, Mastery, Positivity, Achievement, and Satisfaction for Wellbeing Scale. [c]DASS-21: Depression, Anxiety, and Stress Scales – 21-item version. [d]HPQ: Health and Work Performance Questionnaire.

For peer review only

For peer review only - http://bmjopen.bmj.com/site/about/guidelines.xhtml

REFERENCES

1. Keyes CLM. The mental health continuum: From languishing to flourishing in life. Journal of Health and Social Behavior. 2002;43(2):207-22.
2. Keyes CLM. Mental illness and/or mental health? Investigating axioms of the complete state model of health. J Consult Clin Psychol. 2005;73(3):539-48.
3. Routledge KM, Burton KL, Williams LM, Harris A, Schofield PR, Clark CR, et al. Shared versus distinct genetic contributions of mental wellbeing with depression and anxiety symptoms in healthy twins. Psychiatry Res. 2016;244:65-70.
4. Mason Stephens J, Iasiello M, Ali K, van Agteren J, Fassnacht DB. The Importance of Measuring Mental Wellbeing in the Context of Psychological Distress: Using a Theoretical Framework to Test the Dual-Continua Model of Mental Health. Behav Sci. 2023;13(5):436.
5. Gatt JM, Burton KL, Schofield PR, Bryant RA, Williams LM. The heritability of mental health and wellbeing defined using COMPAS-W, a new composite measure of wellbeing. Psychiatry Res. 2014;219(1):204-13.
6. Seligman ME, Steen TA, Park N, Peterson C. Positive psychology progress: empirical validation of interventions. Am Psychol. 2005;60(5):410-21.
7. van Agteren J, Iasiello M, Lo L, Bartholomaeus J, Kopsaftis Z, Carey M, et al. A systematic review and meta-analysis of psychological interventions to improve mental wellbeing. Nat Hum Behav. 2021;5:631-52.
8. Fairburn CG, Patel V. The impact of digital technology on psychological treatments and their dissemination. Behav Res Ther. 2017;88:19-25.
9. Torous J, Staples P, Onnela JP. Realizing the potential of mobile mental health: new methods for new data in psychiatry. Curr Psychiatry Rep. 2015;17:61.
10. Badesha K, Wilde S, Dawson DL. Mental health mobile app use to manage psychological difficulties: an umbrella review. Mental Health Review Journal. 2022;27(3):241-80.
11. Linardon J, Cuijpers P, Carlbring P, Messer M, Fuller-Tyszkiewicz M. The efficacy of app-supported smartphone interventions for mental health problems: a meta-analysis of randomized controlled trials. World Psychiatry. 2019;18(3):325-36.
12. Leech T, Dorstyn D, Taylor A, Li W. Mental health apps for adolescents and young adults: A systematic review of randomised controlled trials. Children and Youth Services Review. 2021;127:106073.
13. Wasil AR, Venturo-Conerly KE, Shingleton RM, Weisz JR. A review of popular smartphone apps for depression and anxiety: Assessing the inclusion of evidence-based content. Behav Res Ther. 2019;123:103498.
14. Wasil AR, Palermo EH, Lorenzo-Luaces L, DeRubeis RJ. Is there an app for that? A review of popular apps for depression, anxiety, and well-being. Cognitive and Behavioral Practice. 2022;29:883-901.
15. Lagan S, D'Mello R, Vaidyam A, Bilden R, Torous J. Assessing mental health apps marketplaces with objective metrics from 29,190 data points from 278 apps. Acta Psychiatr Scand. 2021;144(2):201-10.
16. Leigh S, Flatt S. App-based psychological interventions: friend or foe? Evid Based Ment Health. 2015;18(4):97-9.
17. Torous J, Vaidyam A. Multiple uses of app instead of using multiple apps - a case for rethinking the digital health technology toolbox. Epidemiol Psychiatr Sci. 2020;29:e100.
18. Howells A, Ivtzan I, Eiroa-Orosa FJ. Putting the 'app' in happiness: A randomised controlled trial of a smartphone-based mindfulness intervention to enhance wellbeing. Journal of Happiness Studies: An Interdisciplinary Forum on Subjective Well-Being. 2016;17:163-85.

For peer review only - http://bmjopen.bmj.com/site/about/guidelines.xhtml

19. Mak WW, Tong AC, Yip SY, Lui WW, Chio FH, Chan AT, et al. Efficacy and Moderation of Mobile App-Based Programs for Mindfulness-Based Training, Self-Compassion Training, and Cognitive Behavioral Psychoeducation on Mental Health: Randomized Controlled Noninferiority Trial. JMIR Ment Health. 2018;5(4):e60.
20. Bakker D, Kazantzis N, Rickwood D, Rickard N. A randomized controlled trial of three smartphone apps for enhancing public mental health. Behav Res Ther. 2018;109:75-83.
21. Ehrlich C, Hennelly SE, Wilde N, Lennon O, Beck A, Messenger H, et al. Evaluation of an artificial intelligence enhanced application for student wellbeing: Pilot randomised trial of the Mind Tutor. International Journal of Applied Positive Psychology. 2023;9:435-54.
22. Gnanapragasam SN, Tinch-Taylor R, Scott HR, Hegarty S, Souliou E, Bhundia R, et al. Multicentre, England-wide randomised controlled trial of the 'Foundations' smartphone application in improving mental health and well-being in a healthcare worker population. Br J Psychiatry. 2023;222(2):58-66.
23. Golec de Zavala A, Keenan O, Ziegler M, Ciesielski P, Wahl JE, Mazurkiewicz M. App-based mindfulness training supported eudaimonic wellbeing during the COVID19 pandemic. Appl Psychol Health Well Being. 2024;16(1):42-59.
24. MacIsaac A, Mushquash AR, Wekerle C. Writing yourself well: Dispositional self-reflection moderates the effect of a smartphone app-based journaling intervention on psychological wellbeing across time. Behaviour Change. 2023;40:297-313.
25. Yuan NP, Brooks AJ, Burke MK, Crocker R, Stoner GM, Cook P, et al. My Wellness Coach: evaluation of a mobile app designed to promote integrative health among underserved populations. Transl Behav Med. 2022;12(6):752-60.
26. van Agteren J, Bartholomaeus J, Steains E, Lo L, Gerace A. Using a technology-based meaning and purpose intervention to improve well-being: A randomised controlled study. Journal of Happiness Studies: An Interdisciplinary Forum on Subjective Well-Being. 2021;22:3571-91.
27. Chilver MR, Gatt JM. Six-Week Online Multi-component Positive Psychology Intervention Improves Subjective Wellbeing in Young Adults. J Happiness Stud. 2022;23:1267-88.
28. Lau N, O'Daffer A, Yi-Frazier JP, Rosenberg AR. Popular Evidence-Based Commercial Mental Health Apps: Analysis of Engagement, Functionality, Aesthetics, and Information Quality. JMIR Mhealth Uhealth. 2021;9(7):e29689.
29. Marshall JM, Dunstan DA, Bartik W. Smartphone psychology: New approaches towards safe and efficacious mobile mental health apps. Professional Psychology: Research and Practice. 2020;51(3):214-22.
30. Chilver MR, Keller AS, Park HRP, Jamshidi J, Montalto A, Schofield PR, et al. Electroencephalography profiles as a biomarker of wellbeing: A twin study. J Psychiatr Res. 2020;126:114-21.
31. Gatt JM, Burton KLO, Routledge KM, Grasby KL, Korgaonkar MS, Grieve SM, et al. A negative association between brainstem pontine grey-matter volume, well-being and resilience in healthy twins. J Psychiatry Neurosci. 2018;43(6):386-95.
32. Jamshidi J, Williams LM, Schofield PR, Park HRP, Montalto A, Chilver MR, et al. Diverse phenotypic measurements of wellbeing: Heritability, temporal stability and the variance explained by polygenic scores. Genes Brain Behav. 2020;19(8):e12694.
33. Park HRP, Quide Y, Schofield PR, Williams LM, Gatt JM. Grey matter covariation and the role of emotion reappraisal in mental wellbeing and resilience after early life stress exposure. Transl Psychiatry. 2022;12:85.
34. Park HRP, Chilver MR, Montalto A, Jamshidi J, Schofield PR, Williams LM, et al. Associations between mental wellbeing and fMRI neural bases underlying responses to positive emotion in a twin sample. Psychol Med. 2023;53(4):1215-23.

For peer review only - http://bmjopen.bmj.com/site/about/guidelines.xhtml

35. Routledge KM, Burton KLO, Williams LM, Harris A, Schofield PR, Clark CR, et al. The shared and unique genetic relationship between mental well-being, depression and anxiety symptoms and cognitive function in healthy twins. Cogn Emot. 2017;31(7):1465-79.
36. Faul F, Erdfelder E, Lang AG, Buchner A. G*Power 3: a flexible statistical power analysis program for the social, behavioral, and biomedical sciences. Behav Res Methods. 2007;39(2):175-91.
37. Faul F, Erdfelder E, Buchner A, Lang AG. Statistical power analyses using G*Power 3.1: tests for correlation and regression analyses. Behav Res Methods. 2009;41(4):1149-60.
38. Linardon J, Fuller-Tyszkiewicz M. Attrition and adherence in smartphone-delivered interventions for mental health problems: A systematic and meta-analytic review. J Consult Clin Psychol. 2020;88(1):1-13.
39. Kessler RC, Andrews G, Colpe LJ, Hiripi E, Mroczek DK, Normand SL, et al. Short screening scales to monitor population prevalences and trends in non-specific psychological distress. Psychol Med. 2002;32:959-76.
40. Brooks RT, Beard J, Steel Z. Factor structure and interpretation of the K10. Psychol Assess. 2006;18(1):62-70.
41. Merson F, Newby J, Shires A, Millard M, Mahoney A. The temporal stability of the Kessler Psychological Distress Scale. Australian Psychologist. 2021;56(1):38-45.
42. Slade T, Grove R, Burgess P. Kessler Psychological Distress Scale: normative data from the 2007 Australian National Survey of Mental Health and Wellbeing. Australian and New Zealand Journal of Psychiatry. 2011;45(4):308-16.
43. Gatt JM, Alexander R, Emond A, Foster K, Hadfield K, Mason-Jones A, et al. Trauma, Resilience, and Mental Health in Migrant and Non-Migrant Youth: An International Cross-Sectional Study Across Six Countries. Front Psychiatry. 2020;10:997.
44. Lovibond SH, Lovibond PF. Manual for the Depression Anxiety Stress Scales. 2nd edition ed. Sydney: Psychology Foundation of Australia; 1995.
45. Lovibond PF, Lovibond SH. The structure of negative emotional states: Comparison of the Depression Anxiety Stress Scales (DASS) with the Beck Depression and Anxiety Inventories. Behaviour Research and Therapy. 1995;33(3):335-43.
46. Osman A, Wong JL, Bagge CL, Freedenthal S, Gutierrez PM, Lozano G. The Depression Anxiety Stress Scales-21 (DASS-21): further examination of dimensions, scale reliability, and correlates. J Clin Psychol. 2012;68(12):1322-38.
47. Zanon C, Brenner RE, Baptista MN, Vogel DL, Rubin M, Al-Darmaki FR, et al. Examining the Dimensionality, Reliability, and Invariance of the Depression, Anxiety, and Stress Scale-21 (DASS-21) Across Eight Countries. Assessment. 2021;28(6):1531-44.
48. Kessler RC, Barber C, Beck A, Berglund P, Cleary PD, McKenas D, et al. The World Health Organization Health and Work Performance Questionnaire (HPQ). J Occup Environ Med. 2003;45(2):156-74.
49. Kessler RC, Ames M, Hymel PA, Loeppke R, McKenas DK, Richling DE, et al. Using the World Health Organization Health and Work Performance Questionnaire (HPQ) to evaluate the indirect workplace costs of illness. J Occup Environ Med. 2004;46(6 Suppl):S23-S37.
50. Curry OS, Rowland LA, Van Lissa CJ, Zlotowitz S, McAlaney J, Whitehouse H. Happy to help? A systematic review and meta-analysis of the effects of performing acts of kindness on the well-being of the actor. Journal of Experimental Social Psychology. 2018;76:320-9.
51. Kirby JN. Compassion interventions: The programmes, the evidence, and implications for research and practice. Psychology and Psychotherapy: Theory, Research and Practice. 2017;90(3):432-55.
52. Laguna M, Mazur Z, Kedra M, Ostrowski K. Interventions stimulating prosocial

For peer review only - http://bmjopen.bmj.com/site/about/guidelines.xhtml

helping behavior: A systematic review. Journal of Applied Social Psychology. 2020;50(11):676-96.
53. O'Brien E, Kassirer S. People are slow to adapt to the warm glow of giving. Psychological Science. 2019;30(2):193-204.
54. Alberti RE, Emmons ML. A manual for assertiveness trainers. New York: Impact; 1990.
55. Shaffer CS, Shapiro J, Sank LI, Coghlan DJ. Positive changes in depression, anxiety, and assertion following individual and group cognitive behavior therapy intervention. Cognitive Therapy and Research. 1981;5(2):149-57.
56. Speed BC, Goldstein BL, Goldfried MR. Assertiveness training: A forgotten evidence-based treatment. Clinical Psychology: Science and Practice. 2018;25(1):e12216.
57. Vagos P, Pereira A. A cognitive perspective for understanding and training assertiveness. European Psychologist. 2016;21(2):109-21.
58. Zappe C, Epstein D. Assertive training. Journal of Psychosocial Nursing and Mental Health Services. 1987;25(8):23-6.
59. de Gelder B, Huis In 'T Veld EMJ. Cultural differences in emotional expressions and body language. In: Chiao JY, Li S, Seligman R, Turner R, editors. The Oxford Handbook of Cultural Neuroscience. New York: Oxford University Press; 2015. p. 223–34.
60. Olszewski A, Panorska A, Gillam SL. Training verbal and nonverbal communication interview skills to adolescents. Communication Disorders Quarterly. 2017;38(4):206-18.
61. Peterson RT. An Examination of the Relative Effectiveness of Training in Nonverbal Communication: Personal Selling Implications. J Market Educ. 2005;27(2):143-50.
62. Smith LW, Delgado RA. Body language: The interplay between positional behavior and gestural signaling in the genus Pan and its implications for language evolution. Am J Phys Anthropol. 2015;157(4):592-602.
63. Gander F, Proyer RT, Ruch W, Wyss T. Strength-based positive interventions: Further evidence for their potential in enhancing well-being and alleviating depression. Journal of Happiness Studies: An Interdisciplinary Forum on Subjective Well-Being. 2013;14:1241-59.
64. Proyer RT, Gander F, Wellenzohn S, Ruch W. Positive psychology interventions in people aged 50-79 years: Long-term effects of placebo-controlled online interventions on well-being and depression. Aging & Mental Health. 2014;18(8):997-1005.
65. Rust T, Diessner R, Reade L. Strengths only or strengths and relative weaknesses? A preliminary study. The Journal of Psychology: Interdisciplinary and Applied. 2009;143(5):465-76.
66. Curry NA, Kasser T. Can coloring mandalas reduce anxiety? Art Therapy. 2005;22(2):81-5.
67. Holt NJ, Furbert L, Sweetingham E. Cognitive and affective benefits of coloring: Two randomized controlled crossover studies. Art Therapy. 2019;36(4):200-8.
68. Store SJ, Jakobsson N. The effect of mandala coloring on state anxiety: A systematic review and meta-analysis. Art Therapy. 2022;39(4):173-81.
69. van der Vennet R, Serice S. Can coloring mandalas reduce anxiety? A replication study. Art Therapy. 2012;29(2):87-92.
70. Hopper SI, Murray SL, Ferrara LR, Singleton JK. Effectiveness of diaphragmatic breathing for reducing physiological and psychological stress in adults: a quantitative systematic review. JBI Database System Rev Implement Rep. 2019;17(9):1855-76.
71. Jerath R, Crawford MW, Barnes VA, Harden K. Self-regulation of breathing as a primary treatment for anxiety. Appl Psychophysiol Biofeedback. 2015;40:107-15.
72. Mandlik GV, Siopis G, Nguyen B, Ding D, Edwards KM. Effect of a single session of yoga and meditation on stress reactivity: A systematic review. Stress Health.

For peer review only - http://bmjopen.bmj.com/site/about/guidelines.xhtml

2023;40(3):e3324.
73. Shao R, Man ISC, Lee TMC. The effect of slow-paced breathing on cardiovascular and emotion functions: A meta-analysis and systematic review. Mindfulness. 2024;15:1-18.
74. Zaccaro A, Piarulli A, Laurino M, Garbella E, Menicucci D, Neri B, et al. How breath-control can change your life: A systematic review on psycho-physiological correlates of slow breathing. Frontiers in Human Neuroscience. 2018;12:353.
75. Hinsz VB, Tomhave JA. Smile and (half) the world smiles with you, frown and you frown alone. Personality and Social Psychology Bulletin. 1991;17(5):586-92.
76. Hwang HC, Matsumoto D. Facial expressions. In: Matsumoto D, Hwang HC, Frank MG, editors. APA Handbook of Nonverbal Communication: American Psychological Association; 2016. p. 257-87.
77. Penton-Voak IS, Munafo MR, Looi CY. Biased facial-emotion perception in mental health disorders: A possible target for psychological intervention? Current Directions in Psychological Science. 2017;26(3):294-301.
78. Strnadelova B, Halamova J, Kanovsky M. Identification of Primary Facial Emotions in Relation to Level of Self-Criticism. Perception. 2019;48(10):948-67.
79. Hartanto A, Kaur M, Kasturiratna KTAS, Quek FYX, Majeed NM. A critical examination of the effectiveness of gratitude intervention on well-Being Outcomes: A within-person experimental daily diary approach. J Posit Psychol. 2023;18(6):942-57.
80. Heckendorf H, Lehr D, Ebert DD, Freund H. Efficacy of an internet and app-based gratitude intervention in reducing repetitive negative thinking and mechanisms of change in the intervention's effect on anxiety and depression: Results from a randomized controlled trial. Behav Res Ther. 2019;119:103415.
81. Kloos N, Austin J, van't Klooster J-W, Drossaert C, Bohlmeijer E. Appreciating the good things in life during the covid-19 pandemic: A randomized controlled trial and evaluation of a gratitude app. Journal of Happiness Studies: An Interdisciplinary Forum on Subjective Well Being. 2022;23:4001-25.
82. Southwell S, Gould E. A randomised wait list-controlled pre-post-follow-up trial of a gratitude diary with a distressed sample. The Journal of Positive Psychology. 2017;12(6):579-93.
83. Tolcher K, Cauble M, Downs A. Evaluating the effects of gratitude interventions on college student well-being. J Am Coll Health. 2022;72(4):1321-5.
84. Gal E, Stefan S, Cristea IA. The efficacy of mindfulness meditation apps in enhancing users' well-being and mental health related outcomes: a meta-analysis of randomized controlled trials. J Affect Disord. 2021;279:131-42.
85. Luberto CM, Shinday N, Song R, Philpotts LL, Park ER, Fricchione GL, et al. A Systematic Review and Meta-analysis of the Effects of Meditation on Empathy, Compassion, and Prosocial Behaviors. Mindfulness. 2018;9:708-24.
86. Montero-Marin J, Garcia-Campayo J, Perez-Yus MC, Zabaleta-Del-Olmo E, Cuijpers P. Meditation techniques v. relaxation therapies when treating anxiety: a meta-analytic review. Psychol Med. 2019;49(13):2118-33.
87. Vancampfort D, Stubbs B, Van Damme T, Smith L, Hallgren M, Schuch F, et al. The efficacy of meditation-based mind-body interventions for mental disorders: A meta-review of 17 meta-analyses of randomized controlled trials. J Psychiatr Res. 2021;134:181-91.
88. Zeng X, Chiu CPK, Wang R, Oei TPS, Leung FYK. The effect of loving-kindness meditation on positive emotions: a meta-analytic review. Front Psychol. 2015;6:1693.
89. Zheng Y, Yan L, Chen Y, Zhang X, Sun W, Lv J, et al. Effects of loving-kindness and compassion meditation on anxiety: A systematic review and meta-analysis. Mindfulness. 2023;14:1021-37.
90. Chen B, Yang T, Xiao L, Xu C, Zhu C. Effects of Mobile Mindfulness Meditation on

For peer review only - http://bmjopen.bmj.com/site/about/guidelines.xhtml

the Mental Health of University Students: Systematic Review and Meta-analysis. J Med Internet Res. 2023;25:e39128.
91. Goldberg SB, Riordan KM, Sun S, Davidson RJ. The empirical status of mindfulness-based interventions: A systematic review of 44 meta-analyses of randomized controlled trials. Perspectives on Psychological Science. 2022;17(1):108-30.
92. Gotink RA, Meijboom R, Vernooij MW, Smits M, Hunink MG. 8-week Mindfulness Based Stress Reduction induces brain changes similar to traditional long-term meditation practice - A systematic review. Brain Cogn. 2016;108:32-41.
93. Johnson BT, Acabchuk RL, George EA, Nardi W, Sun S, Salmoirago-Blotcher E, et al. Mental and physical health impacts of mindfulness training for college undergraduates: A systematic review and meta-analysis of randomized controlled trials. Mindfulness. 2023;14:2077-96.
94. Schwartz K, Ganster FM, Tran US. Mindfulness-Based Mobile Apps and Their Impact on Well-Being in Nonclinical Populations: Systematic Review of Randomized Controlled Trials. J Med Internet Res. 2023;25:e44638.
95. Tan ZYA, Wong SH, Cheng LJ, Lau ST. Effectiveness of mobile-based mindfulness interventions in improving mindfulness skills and psychological outcomes for adults: A systematic review and meta-regression. Mindfulness. 2022;13:2379-95.
96. Taylor H, Strauss C, Cavanagh K. Can a little bit of mindfulness do you good? A systematic review and meta-analyses of unguided mindfulness-based self-help interventions. Clin Psychol Rev. 2021;89:102078.
97. Gloster AT, Walder N, Levin ME, Twohig MP, Karekla M. The empirical status of acceptance and commitment therapy: A review of meta-analyses. Journal of Contextual Behavioral Science. 2020;18:181-92.
98. Serowik KL, Khan AJ, LoCurto J, Orsillo SM. The conceptualization and measurement of values: A review of the psychometric properties of measures developed to inform values work with adults. Journal of Psychopathology and Behavioral Assessment. 2018;40:615-35.
99. Stenhoff A, Steadman L, Nevitt S, Benson L, White RG. Acceptance and commitment therapy and subjective wellbeing: A systematic review and meta-analyses of randomised controlled trials in adults. Journal of Contextual Behavioral Science. 2020;18:256-72.
100. Witteman HO, Ndjaboue R, Vaisson G, Dansokho SC, Arnold B, Bridges JFP, et al. Clarifying Values: An Updated and Expanded Systematic Review and Meta-Analysis. Med Decis Making. 2021;41(7):801-20.
101. Han A, Kim TH. Effects of internet-delivered behavioral activation on individuals with depressive symptoms: A systematic review and meta-analysis. J Psychiatr Res. 2022;152:104-18.
102. Huguet A, Miller A, Kisely S, Rao S, Saadat N, McGrath PJ. A systematic review and meta-analysis on the efficacy of Internet-delivered behavioral activation. J Affect Disord. 2018;235:27-38.
103. Layous K, Chancellor J, Lyubomirsky S. Positive activities as protective factors against mental health conditions. J Abnorm Psychol. 2014;123(1):3-12.
104. Ong AD, Lee S. Variety in pleasant activities is associated with improved mental health: Evidence from two national samples of U.S. adults. Affective Science. 2023;5:90-8.
105. Panaite V, Yoon S, Devendorf AR, Kashdan TB, Goodman FR, Rottenberg J. Do positive events and emotions offset the difficulties of stressful life events? A daily diary investigation of depressed adults. Personality and Individual Differences. 2022;186(Part A):111379.
106. Spates CR, Padalino R, Hale AC, Germain CS, Nimmo K, Kohler R. A review of

For peer review only - http://bmjopen.bmj.com/site/about/guidelines.xhtml

web-based technology in behavioural activation. Clinical Psychologist. 2016;20(1):27-35.
107. Vanderkruik R, Whisman MA. A cross-cultural examination of pleasant events and depressive symptoms. Journal of Clinical Psychology. 2021;77:1384-93.
108. Bryant FB, Smart CM, King SP. Using the past to enhance the present: Boosting happiness through positive reminiscence. Journal of Happiness Studies: An Interdisciplinary Forum on Subjective Well-Being. 2005;6:227-60.
109. Hallford DJ, Hardgrove S, Sanam M, Oliveira S, Pilon M, Duran T. Remembering for resilience: Brief cognitive-reminiscence therapy improves psychological resources and mental well-being in young adults. Applied Psychology: Health and Well Being. 2022;14:1004-21.
110. Miguel-Alvaro A, Guillen AI, Contractor AA, Crespo M. Positive memory intervention techniques: a scoping review. Memory. 2021;29(6):793-810.
111. Tam W, Poon SN, Mahendran R, Kua EH, Wu XV. The effectiveness of reminiscence-based intervention on improving psychological well-being in cognitively intact older adults: A systematic review and meta-analysis. Int J Nurs Stud. 2021;114:103847.
112. Westerhof GJ, Bohlmeijer E, Webster JD. Reminiscence and mental health: A review of recent progress in theory, research and interventions. Ageing & Society. 2010;30:697-721.
113. Dickens LR, Robins RW. Pride: A meta-analytic project. Emotion. 2022;22(5):1071-87.
114. Ding K, Liu J. Comparing gratitude and pride: evidence from brain and behavior. Cogn Affect Behav Neurosci. 2022;22:1199-214.
115. Lin D, Bi J, Zhang X, Zhu F, Wang Y. Successful emotion regulation via cognitive reappraisal in authentic pride: Behavioral and event-related potential evidence. Front Hum Neurosci. 2022;16:983674.
116. Van Doren N, Tharp JA, Johnson SL, Staudenmaier PJ, Anderson C, Freeman MA. Perseverance of effort is related to lower depressive symptoms via authentic pride and perceived power. Personality and Individual Differences. 2019;137:45-9.
117. Weidman AC, Tracy JL, Elliot AJ. The benefits of following your pride: Authentic pride promotes achievement. Journal of Personality. 2016;84(5):607-22.
118. Allen AB, Leary MR. Self-compassion, stress, and coping. Social and Personality Psychology Compass. 2010;4(2):107-18.
119. Barnard LK, Curry JF. Self-compassion: Conceptualizations, correlates, & interventions. Review of General Psychology. 2011;15(4):289-303.
120. Ferrari M, Hunt C, Harrysunker A, Abbott MJ, Beath AP, Einstein DA. Self-compassion interventions and psychosocial outcomes: A meta-analysis of RCTs. Mindfulness. 2019;10:1455-73.
121. Neff KD. The development and validation of a scale to measure self-compassion. Self and Identity. 2003;2(3):223-50.
122. Neff KD, Kirkpatrick KL, Rude SS. Self-compassion and adaptive psychological functioning. Journal of Research in Personality. 2007;41(1):139-54.
123. Zessin U, Dickhauser O, Garbade S. The relationship between self-compassion and well-being: A meta-analysis. Applied Psychology: Health and Well-Being. 2015;7(3):340-64.
124. Bjorvatn K, Ekstrom M, Garcia Pires AJ. Setting goals for keystone habits improves labor market prospects and life satisfaction for unemployed youth: Experimental evidence from Norway. Journal of Economic Behavior & Organization. 2021;188:1109-23.
125. Bovend'Eerdt TJH, Botell RE, Wade DT. Writing SMART rehabilitation goals and achieving goal attainment scaling: a practical guide. Clin Rehabil. 2009;23(4):352-61.
126. Cairns AJ, Kavanagh DJ, Dark F, McPhail SM. Goal setting improves retention in youth mental health: a cross-sectional analysis. Child Adolesc Psychiatry Ment Health. 2019;13:31.

For peer review only - http://bmjopen.bmj.com/site/about/guidelines.xhtml

127. Carrillo A, Rubio-Aparicio M, Molinari G, Enrique A, Sanchez-Meca J, Banos RM. Effects of the Best Possible Self intervention: A systematic review and meta-analysis. PLoS One. 2019;14(9):e0222386.
128. Elzubeir K, Dye S. Can amount and duration of seclusion be reduced in psychiatric intensive care units by agreeing SMART goals with patients? Journal of Psychiatric Intensive Care. 2017;13(2):109-16.
129. Epton T, Currie S, Armitage CJ. Unique effects of setting goals on behavior change: Systematic review and meta-analysis. Journal of Consulting and Clinical Psychology. 2017;85(12):1182-98.
130. Weintraub J, Cassell D, DePatie TP. Nudging flow through 'SMART' goal setting to decrease stress, increase engagement, and increase performance at work. Journal of Occupational and Organizational Psychology. 2021;94:230-58.
131. Batt-Rawden SA, Chisolm MS, Anton B, Flickinger TE. Teaching empathy to medical students: An updated, systematic review. Academic Medicine. 2013;88(8):1171-7.
132. Bell H. Creative interventions for teaching empathy in the counseling classroom. Journal of Creativity in Mental Health. 2018;13(1):106-20.
133. D'Souza PC, Rasquinha SL, D'Souza TL, Jain A, Kulkarni V, Pai K. Effect of a single-session communication skills training on empathy in medical students. Academic Psychiatry. 2020;44:289-94.
134. Davis JR, Gold GJ. An examination of emotional empathy, attributions of stability, and the link between perceived remorse and forgiveness. Personality and Individual Differences. 2011;50(3):392-7.
135. Riess H, Kelley JM, Bailey RW, Dunn EJ, Phillips M. Empathy training for resident physicians: A randomized controlled trial of a neuroscience-informed curriculum. Journal of General Internal Medicine. 2012;27(10):1280-6.
136. Carver CS. You want to measure coping but your protocol's too long: Consider the Brief COPE. International Journal of Behavioral Medicine. 1997;4(1):92-100.
137. Carver CS, Scheier MF, Weintraub JK. Assessing coping strategies: A theoretically based approach. Journal of Personality and Social Psychology. 1989;56(2):267-83.
138. Fischer R, Scheunemann J, Moritz S. Coping strategies and subjective well-being: Context matters. Journal of Happiness Studies: An Interdisciplinary Forum on Subjective Well-Being. 2021;22:3413-34.
139. Meng X, D'Arcy C. Coping strategies and distress reduction in psychological well-being? A structural equation modelling analysis using a national population sample. Epidemiology and Psychiatric Sciences. 2016;25(4):370-83.
140. Skinner EA, Edge K, Altman J, Sherwood H. Searching for the structure of coping: A review and critique of category systems for classifying ways of coping. Psychological Bulletin. 2003;129(2):216-69.

For peer review only - http://bmjopen.bmj.com/site/about/guidelines.xhtml

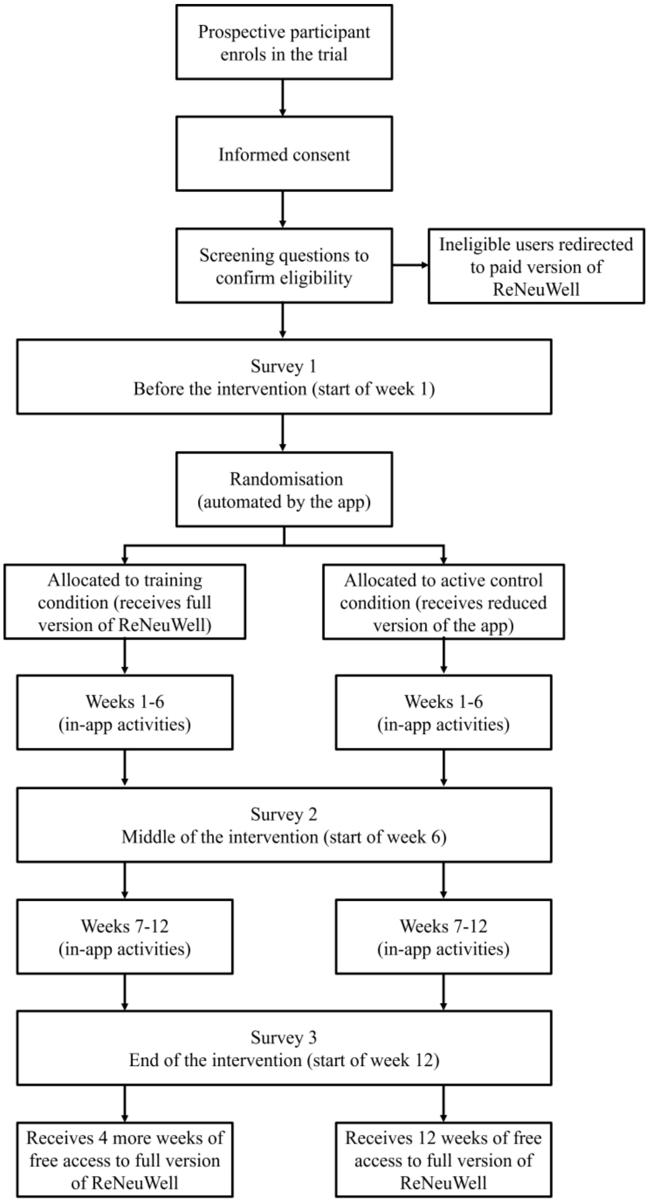

Figure 1: Study timeline.

1632x3023mm (38 x 38 DPI)

Supplementary Materials

for

The ReNeuWell mental wellbeing app:

Protocol for a randomised controlled trial

Luke A. Egan[1] and Justine M. Gatt[1,2,3*]

[1] Centre for Wellbeing, Resilience and Recovery, Neuroscience Research Australia, Randwick, NSW, Australia.

[2] School of Psychology, University of New South Wales, Kensington, NSW, Australia.

[3] Black Dog Institute, Randwick, NSW, Australia

***Corresponding author:** Associate Professor Justine Gatt; Phone: +612 9399 1812; Postal address: Neuroscience Research Australia, Barker St, Randwick, NSW 2031, Australia; E-mail address: j.gatt@unsw.edu.au

For peer review only

Supplementary Materials

Screening questions (in addition to K10)

1. How old are you?

2. Do you understand written English?

3. What country do you currently live in?
   - ☐ Australia
   - ☐ Canada
   - ☐ Ireland
   - ☐ New Zealand
   - ☐ USA
   - ☐ UK
   - ☐ Other: ___________________________

4. Do you currently suffer from a psychiatric or psychological condition? (e.g., anxiety disorder, depression, bipolar disorder, PTSD, phobia, psychosis, schizophrenia, eating disorder etc)

For peer review only

DEMOGRAPHICS

1	What is your age (in years)?	
2	Gender	☐ Male<br>☐ Female<br>☐ Non-binary / Other
3	What country do you currently live in?	☐ Australia<br>☐ Canada<br>☐ Ireland<br>☐ New Zealand<br>☐ USA<br>☐ UK
4	What state/ province/ county do you currently live in?	________________________________
5	What is your current marital status?	☐ Single<br>☐ Have a boyfriend/girlfriend<br>☐ Married / De facto (living with partner)<br>☐ Separated / Divorced<br>☐ Widowed
6	Height	________ cm or ________ feet/inches
7	Weight	________ kg or _________ pounds
8	Highest education completed	☐ High School<br>☐ Trade Certificate<br>☐ Graduate diploma or graduate certificate<br>☐ Undergraduate degree (Bachelor)<br>☐ Postgraduate degree (Master / PhD)
9	What is your current employment status?	☐ Full-time<br>☐ Part-time<br>☐ Studying and working<br>☐ Studying only<br>☐ Unemployed and looking for work<br>☐ Not doing paid work and not looking for work
10	What is your current main occupation?	________________________________
11	What is your role in your organization? (e.g., employee, manager, executive manager)	________________________________

For peer review only - http://bmjopen.bmj.com/site/about/guidelines.xhtml

12	Please think of the following 10-point scale like a ladder, representing where people stand in their society. People define society in different ways; please define it in whatever way is most meaningful to you.<br>At the top of the scale ("10") are the people who have the highest standing in their society, and at the bottom ("1") are the people who have the lowest standing in their society.<br>Where would you place yourself on this scale?	☐ 10. Highest standing in society<br>☐ 9.<br>☐ 8.<br>☐ 7.<br>☐ 6.<br>☐ 5.<br>☐ 4.<br>☐ 3.<br>☐ 2.<br>☐ 1. Lowest standing in society

HEALTH

1	Have you ever been diagnosed with a psychiatric or psychological condition? (e.g., eating disorder, anxiety disorder, depression, social phobia, PTSD)?	☐ Yes<br>☐ No<br>☐ Prefer not to answer
If "No" or "Prefer not to answer", finish this section.		
2	Which psychiatric disorders have you been diagnosed with? Please tick all that apply.	☐ Eating Disorder<br>☐ Generalized Anxiety Disorder<br>☐ Panic Disorder<br>☐ Post-traumatic stress disorder (PTSD)<br>☐ Social Phobia / Social Anxiety Disorder<br>☐ Major Depression<br>☐ Bipolar Disorder (Manic Depression)<br>☐ Schizophrenia<br>☐ Obsessive Compulsive Disorder<br>☐ Personality Disorder (e.g. Borderline Personality Disorder)<br>☐ Tourette's Disorder<br>☐ Other:<br>_______________________________
3	What age were you when you were first diagnosed?	____________ years old
4	For how long did you suffer from this condition?	☐ Less than 1 year<br>☐ 1 – 3 years<br>☐ 3 – 5 years<br>☐ Ongoing

For peer review only

For peer review only - http://bmjopen.bmj.com/site/about/guidelines.xhtml

5	Did you receive treatment for this condition? Please tick all that apply.	☐ No treatment<br>☐ I received behavioural therapy (e.g., CBT):<br>___________ (please specify)<br>☐ I received medication treatment (e.g., antidepressants):<br>___________ (please specify)<br>☐ Alternative therapies (e.g., homeopathy, naturopathy):<br>___________ (please specify)

LIFESTYLE

1	On average, how many cups of caffeinated drinks (coffee, coke or energy drinks) do you drink per day?	☐ None<br>☐ 1 – 2 cups<br>☐ 3 – 4 cups<br>☐ Over 5 cups
2	Has there ever been a time when you smoked cigarettes regularly? That is, at least one cigarette per day for 3 months or longer?	☐ Yes<br>☐ No
If "No", go to question #9.		
3	How old were you when you first started smoking cigarettes regularly?	__________ years old
4	In total, how many years have you smoked cigarettes regularly?	__________ years
5	Over the time you smoked regularly, how many cigarettes on average did you smoke per day?	☐ 1-5 cigarettes per day<br>☐ 6-10 cigarettes per day<br>☐ 11-20 cigarettes per day<br>☐ 21-30 cigarettes per day<br>☐ More than a pack a day
6	Are you currently smoking cigarettes regularly?	☐ Yes<br>☐ No
If "Yes", go to question #7 then question #9.<br>If "No", go to question #8.		
7	Over the last year, how many cigarettes on average did you smoke per day?	☐ 1-5 cigarettes per day<br>☐ 6-10 cigarettes per day<br>☐ 11-20 cigarettes per day<br>☐ 21-30 cigarettes per day<br>☐ More than a pack a day

For peer review only - http://bmjopen.bmj.com/site/about/guidelines.xhtml

For peer review only

For peer review only - http://bmjopen.bmj.com/site/about/guidelines.xhtml

8	When did you stop smoking cigarettes regularly?	☐ I stopped smoking in the past 3 months<br>☐ I stopped smoking in the past 3-6 months<br>☐ I stopped smoking in the past 6-12 months<br>☐ I stopped smoking last year<br>☐ I stopped smoking when I was about: _____ years old
9	How often do you have a drink containing alcohol?	☐ Never<br>☐ Monthly or less<br>☐ 2-4 times a month<br>☐ 2-3 times a week<br>☐ 4 or more times a week
10	How many drinks containing alcohol do you have on a typical day when you are drinking?	☐ 1 or 2<br>☐ 3 or 4<br>☐ 5 or 6<br>☐ 7 to 9<br>☐ 10 or more
11	How often do you have 6 or more drinks on one occasion?	☐ Never<br>☐ Less than monthly<br>☐ Monthly<br>☐ Weekly<br>☐ Daily or almost daily
12	On average, how many times a week do you exercise (e.g., strength training, aerobic exercise, or activities such as running, swimming, strenuous walking or bike riding) for at least 30 minutes at a time?	☐ None<br>☐ 1 – 2 times<br>☐ 3 – 4 times<br>☐ 5 – 6 times<br>☐ Daily
13	On average, how many times a week do you eat fruit and vegetables?	☐ None<br>☐ 1 – 2 times<br>☐ 3 – 4 times<br>☐ 5 – 6 times<br>☐ Daily
14	How many hours of sleep do you usually get per night?	☐ Less than 3 hours<br>☐ 3 – 5 hours<br>☐ 6 – 8 hours<br>☐ Over 8 hours

For peer review only - http://bmjopen.bmj.com/site/about/guidelines.xhtml

APP SATISFACTION SURVEY

1. Overall, how important is it to you to improve your own wellbeing?

☐ Very important
☐ Moderately important
☐ Neutral
☐ Slightly important
☐ Not important

2. How many apps have you previously downloaded or used that aim to improve your wellbeing (including meditation and mindfulness apps)?

☐ None previously. This is my first time
☐ 1-2 apps previously
☐ 3 or more apps previously

3. Have you previously attended group classes like yoga and meditation?

☐ No, never
☐ Yes, I have attended a few classes previously
☐ Yes, I attend yoga and/or meditative classes on a regular basis

4. Overall, how helpful do you feel the app was in increasing your mental wellbeing (e.g., mental health, positivity, emotional health)?

☐ Very helpful
☐ Quite a lot
☐ Somewhat
☐ A little
☐ Not at all helpful

5. Overall, how helpful do you feel the app was in increasing your physical wellbeing (e.g., vigour, fitness, sleep)?

☐ Very helpful
☐ Quite a lot
☐ Somewhat
☐ A little
☐ Not at all helpful

6. When using the app, how easy was it to find core features and navigate the app?

☐ Very easy
☐ Easy
☐ Neutral
☐ Difficult
☐ Very difficult

For peer review only - http://bmjopen.bmj.com/site/about/guidelines.xhtml

7. Do you see the value in using the app?

- ☐ Yes, absolutely
- ☐ Yes, mostly
- ☐ Sometimes
- ☐ Not always
- ☐ Not at all

8. What is the likelihood you will continue using the app for your own wellbeing?

- ☐ Extremely likely
- ☐ Likely
- ☐ Neutral
- ☐ Unlikely
- ☐ Extremely unlikely

9. Do you prefer to use this app over other wellbeing apps?

- ☐ Yes
- ☐ No

If no, what other apps do you prefer and why? ________________________

10. What did you like the most about the app? ________________________

11. What activity did you like the most? ________________________

12. What did you dislike about the app? ________________________

13. What other information would you like to see in the app? ________________________

14. Other suggestions to improve the experience? ________________________

For peer review only

Adverse Event Assessment

Thank you for taking the time to share your answers and feedback. Your responses are anonymous and confidential, and the survey responses will be used to help develop our app further.

If any aspect of this survey or app caused you distress, please describe in the space below, including which items or features (otherwise you can leave it blank):

____
____
____
____

For peer review only - http://bmjopen.bmj.com/site/about/guidelines.xhtml

BMJ Open

The ReNeuWell mental wellbeing app: Protocol for a randomised controlled trial

Journal:	*BMJ Open*
Manuscript ID	bmjopen-2024-094557.R1
Article Type:	Protocol
Date Submitted by the Author:	20-Feb-2025
Complete List of Authors:	Egan, Luke; Neuroscience Research Australia, Centre for Wellbeing, Resilience and Recovery<br>Gatt, Justine; Neuroscience Research Australia, Centre for Wellbeing, Resilience and Recovery; University of New South Wales - Kensington Campus, School of Psychology
**Primary Subject Heading**:	Mental health
Secondary Subject Heading:	Mental health
Keywords:	MENTAL HEALTH, Psychosocial Intervention, Adult psychiatry < PSYCHIATRY, Internet, Digital Technology, Mindfulness

SCHOLARONE™
Manuscripts

The ReNeuWell mental wellbeing app:

Protocol for a randomised controlled trial

Luke A. Egan[1] and Justine M. Gatt[1,2*]

[1] Centre for Wellbeing, Resilience and Recovery, Neuroscience Research Australia, Randwick, NSW, Australia.

[2] School of Psychology, University of New South Wales, Kensington, NSW, Australia.

***Correspondence:** Associate Professor Justine Gatt; Ph: +612 9399 1812; Postal address: Neuroscience Research Australia, Barker St, Randwick, NSW 2031, Australia; Email: j.gatt@unsw.edu.au

Word count:

Abstract: 438 words

Manuscript: 5638 words

Tables: 2 tables (193 words)

Figures: 1 figure

Supplementary Materials: 1 file (PDF document, 2687 words)

References: 4576 words

ORCID IDs

Luke A. Egan[1] ORCID ID: 0000-0003-0544-8851

Justine M. Gatt[1,2*] ORCID ID: 0000-0002-9276-6358

For peer review only - http://bmjopen.bmj.com/site/about/guidelines.xhtml

ABSTRACT

Introduction: The field of mental wellbeing interventions includes numerous studies of smartphone app-based programs, but there is a *research-to-retail gap* where many studies pertain to apps that are not publicly available, not used as standalone programs, or not tested in the general population, and many publicly available apps (or their proprietary in-app measures) have yet to be submitted to empirical testing. Furthermore, few wellbeing apps offer multi-component interventions, despite such interventions having demonstrated efficacy outside the smartphone context. In response to these openings in the literature and marketplace, we have developed ReNeuWell, an iPhone app designed to measure the user's mental wellbeing (via the validated COMPAS-W scale) and improve their wellbeing via a personalised, multi-component program of activities informed by the peer-reviewed evidence base. This paper describes the protocol for the pre-registered randomised controlled trial (RCT) of ReNeuWell, to test the app in adult participants from the general population of Apple App Store users. It is hypothesized that ReNeuWell users will experience significant increases in mental wellbeing and decreases in mental distress over the 6–12-week trial period, relative to users of an active control version of the app.

Methods and analysis: The RCT will recruit participants from Apple store users who choose to download ReNeuWell in the normal course of browsing the marketplace. Following consent, the app will randomly allocate participants to receive either the full version of the app or the active control version. The full version assesses the user's wellbeing via the validated COMPAS-W scale, provides feedback on their wellbeing across six dimensions, and creates a personalised schedule of daily positive psychology activities designed to enhance wellbeing along each dimension. Participants will be instructed to use the app for at least 10 minutes (at least one activity) per day for the first 6 weeks, and as they

For peer review only - http://bmjopen.bmj.com/site/about/guidelines.xhtml

wish for the following 6 weeks. Trial outcomes will be measured via in-app surveys administered in weeks 1, 6, and 12. Data collection will begin when the app is officially launched on the Apple store. Data will be analysed using linear mixed models to estimate condition-by-time interaction effects on the primary and secondary outcomes, and to assess whether any such effects are themselves moderated by other key variables.

Ethics and dissemination: This protocol has been approved by the Human Research Ethics Committee of the University of New South Wales (reference number: HC210302). Trial outcomes will be published in accordance with the pre-registered protocol described here, both in the peer-reviewed literature and on the registry website.

Registration: This protocol was pre-registered with the Australian New Zealand Clinical Trials Registry (registration approved on 4 August 2021; trial ID number: ACTRN12621001014842p).

Strengths and limitations of this study

- ReNeuWell is a newly developed, publicly available iPhone app that addresses gaps in both the literature and the market, incorporating a validated wellbeing measure (the COMPAS-W scale) and a personalised, multi-component program of evidence-based activities and resources.
- The RCT of ReNeuWell accounts for the placebo effect by offering an active control condition, and includes both a primary endpoint (6 weeks after baseline measures) and a secondary endpoint (12 weeks), allowing follow-up measurements of possible sustained benefits.
- The primary outcomes encompass both mental wellbeing and psychological distress (depression, anxiety, stress), which reflects the modern conception of mental health as comprising both minimal mental illness and optimal mental wellbeing.

For peer review only - http://bmjopen.bmj.com/site/about/guidelines.xhtml

For peer review only

- The participant screening process excludes those with moderate-to-severe distress, which may increase the likelihood of ceiling effects (for wellbeing outcomes) and floor effects (for distress outcomes).
- The study period encompasses a follow-up measure at 12 weeks after baseline, but if efficacy is demonstrated, this period could be expanded to longer time-frames to assess maintenance of effects.

For peer review only

For peer review only - http://bmjopen.bmj.com/site/about/guidelines.xhtml

Introduction

Optimal mental health is defined not only by minimal symptoms of mental illness but also by high levels of mental wellbeing [1-4]. Low wellbeing, even without accompanying mental illness symptoms, can lead to poor psychosocial outcomes [1, 5], necessitating psychological programs that target wellbeing specifically, even in non-clinical populations [6, 7]. Such interventions are increasingly offered via digital technologies such as websites and smartphone apps [8-10], some of which have been empirically tested [11, 12], but there remains the *research-to-retail gap*: much of the research pertains to apps that are not publicly available on app stores, have not been tested in the general population of app users, and/or are not offered as standalone programs [13, 14], which raises questions of ecological validity. Some studies even pertain to apps that were not given a product name, precluding other researchers from following up on the results. On the market side of the gap, it is common to find long-standing apps whose product descriptions make claims of improved mental health or wellbeing despite little-to-no empirical evidence [10, 12, 15-17], and apps that incorporate proprietary psychological measures (e.g., happiness questionnaires) that have not been validated in the peer-reviewed literature. Therefore, despite the large number of mental health apps that have been released over the years [10, 14, 15], there are still openings in the market for new, standalone apps that are both evidence-based and publicly available, as well as openings in the literature for peer-reviewed studies of such apps, incorporating established mental health measures.

The research-to-retail gap can be illustrated by considering the studies included in Linardon et al.'s recent meta-analysis of randomized controlled trials (RCTs) of app-supported mental health interventions [11]. The meta-analysis incorporated 66 effects across 46 studies, finding that app-supported interventions can lead to small-to-moderate reductions in depression, anxiety, and stress; and small-to-moderate increases in mental wellbeing and

For peer review only - http://bmjopen.bmj.com/site/about/guidelines.xhtml

quality of life. However, few of the 46 papers described interventions that resembled the *typical experience* of downloading a publicly available app from an app store and using it as a *standalone* program without any accompanying resources or interactions [14]. Across the 66 trials, only 14 publicly available mental health apps were examined, and of those apps, only Headspace was evaluated in more than one study. Of the three studies that examined Headspace, only one [18] involved a sample from the general population of adults to which the app is marketed. Only four other apps – Living with Heart [19], MoodKit, MoodMission, and MoodPrism – were studied in samples from the general population, and the latter three were evaluated in the same sample from a single study [20]. Since Linardon et al.'s [11] meta-analysis, additional RCTs of mental wellbeing apps have been published (for example, [21-27]), and while it is beyond the scope of this paper to provide an exhaustive literature review, we have noted that few of these more recent trials were conducted in the general population of app store users (e.g., van Agteren et al. [26] sampled from the general adult population, although the participants were not recruited "naturally" through self-initiated downloads from an app store). Thus, there is actually little research on app-based wellbeing interventions within ecologically valid contexts. This is not a criticism of any individual study. For example, it is perfectly worthwhile to test whether a given mental health app benefits members of a subpopulation (e.g., people with diagnosed major depressive disorder); we are simply noting that such studies are less relevant to our understanding of how such apps affect users in general, to whom all the top mental health apps are marketed [14].

Although the evidence on app-based wellbeing interventions is relatively limited, it is nonetheless encouraging. The available research suggests that such interventions can produce significant mental health benefits in terms of both wellbeing and illness/symptom levels. For example, there is evidence that wellbeing apps can lead to increases in mental wellbeing [19, 20, 22, 23], life satisfaction [26], positive affect [18, 26], and self-compassion [19, 28];

For peer review only - http://bmjopen.bmj.com/site/about/guidelines.xhtml

improvements in affective balance [23, 27]; reductions in depression [18, 20], anxiety [28], stress [23, 27, 28], and other psychiatric symptoms [22]; and improved sleep [22, 28]. Of course, for a wellbeing app to have broad impact, it must be not only effective but also utilised by a sufficiently large number of people. The MIND website (Mobile-health Index & Navigation Database; see [15, 29]) provides a public catalogue of mental health apps, informed by the American Psychiatric Association's App Evaluation Model. On 10 February 2025, we searched this catalogue for the term "wellbeing" and related terms such as "wellness", "happiness", "satisfaction", and "positive", retrieving 21 results. After excluding apps that were not designed to increase mental wellbeing, not designed for the general public, and/or not designed to be standalone, self-guided programs, we were left with 9 apps whose download numbers were publicly available on the Google Play store (the Apple store does not display such data). All 9 apps had been downloaded at least 100,000 times. Although downloading an app does not imply that the user will actually use the app extensively [30], these figures illustrate the demand for app-based wellbeing interventions and the feasibility of their delivery at scale.

For a new wellbeing app to be potentially beneficial, it should offer something beyond the contents of existing apps, while being grounded in an established model of mental wellbeing. A recent narrative review [14] examined the top 20 mental health apps on the Apple and Google stores, which together accounted for 98% of monthly active users (MAUs, i.e., unique individuals who used a mental health app at least once in the one-month reference period). Of these apps, 18 provided unguided, standalone programs, most of which fell into one of three categories: mindfulness/meditation, journaling/self-monitoring, or conversing with an artificial intelligence (AI) chatbot. The top two mindfulness/meditation apps – Headspace and Calm – together accounted for approximately 70% of MAUs. Clearly, those seeking mental health support via smartphone apps either prefer mindfulness/meditation

For peer review only

For peer review only - http://bmjopen.bmj.com/site/about/guidelines.xhtml

exercises or have few alternatives. The latter is plausible: Lagan et al.'s recent review of 278 mental health apps [15] found that the three most common activities (provided by hundreds of different apps) were self-monitoring, journaling, and mindfulness exercises. In contrast, exercises from evidence-based therapies, such as cognitive behavioural therapy (CBT) or acceptance and commitment therapy (ACT), were offered by far fewer apps (<60 for CBT and <20 for ACT). Thus, there may be unmet demand for new apps that provide activities and resources not included in many (if any) of the current offerings. The positive psychology literature describes numerous exercises and strategies to raise mental wellbeing whose efficacy has been demonstrated in non-app interventions [6, 7, 31]; these are prime candidates for conversion to app-based formats where possible. Lagan et al.'s [15] review also highlighted "the lack of comprehensive apps that would facilitate multiple diverse uses" (p. 8). Although a user could download multiple apps to cover a range of activities, there are numerous drawbacks of this approach [17], including the obvious inconvenience, the possibility of receiving contradictory notifications or advice from two or more apps, and (with paid apps) the expense.

Furthermore, van Agteren et al.'s recent meta-analysis of mental wellbeing interventions [7] concluded that the two kinds of intervention most consistently associated with positive outcomes were mindfulness-based activities and *multi-component* positive psychology interventions (comprising two or more positive psychology techniques in a single program). Although this meta-analysis was not limited to app-based or digital interventions, it clearly suggests that multiple activities should be considered in programs to improve mental wellbeing. Despite this, a recent review of 19 popular mental health apps [32] found that 8 of the apps offered only a single evidence-based treatment component (e.g., meditation only), with an average of 2.32 components per app across all 19 apps, none of which offered more than 5 components (for comparison, van Agteren et al.'s meta-analysis covered over 15 types

For peer review only - http://bmjopen.bmj.com/site/about/guidelines.xhtml

of positive psychology intervention). Moreover, Marshall et al. [33] have observed that many mental health apps "only offer singular or novelty interventions that do not qualify as comprehensive therapeutic treatments and/or diagnostic instruments" (p. 217).

Given the preceding considerations, we have developed the forthcoming iPhone app "ReNeuWell" (a portmanteau of "Resilience, Neuroscience, Wellbeing"), which may address the opening for a standalone, publicly available, multi-component wellbeing app for the general adult population. ReNeuWell comprises psychoeducational materials, positive psychology exercises, and numerous other activities and resources, with the aim of increasing mental wellbeing in users. We included as many evidence-based activities and resources as possible, within the inherent limitations of app-based delivery as well as budgetary and logistical constraints. To track each user's wellbeing and provide them with a personalised program, ReNeuWell incorporates a comprehensive measure of mental wellbeing – the COMPAS-W scale [5] – which has been validated in numerous prior studies with regard to both its psychometric properties and various correlates including health behaviours, work productivity, genetics, neural activity, and cognitive functioning [3, 5, 34-39]. Indeed, the entire app is grounded in the COMPAS-W model of wellbeing. Each activity is designed to target one of the six dimensions of wellbeing measured by the COMPAS-W, and the psychoeducational materials educate the user on the six dimensions and how each contributes to total wellbeing. In short, ReNeuWell addresses key limitations of many previous app-based mental health interventions. It offers a multi-component program, it tracks user outcomes via a validated wellbeing measure, its activities are explicitly based on the peer-reviewed literature, and it allows our proposed RCT to have greater ecological validity, because the app provides a standalone, self-guided intervention for smartphone users in the general adult population, who will access it via self-initiated downloads from the app store.

Given our target population, it is worth noting both that there is a need for greater

For peer review only - http://bmjopen.bmj.com/site/about/guidelines.xhtml

mental wellbeing in the general adult population and that this outcome can indeed be achieved via the kinds of positive psychological techniques offered by our app. As mentioned above, mental health requires both minimal illness and optimal wellbeing. These domains comprise *dual continua*, each of which shares approximately 28% of its variance with the other [2]. Thus, treating mental illness does not imply that mental wellbeing will necessarily improve. A substantial body of research has shown that even among those without symptoms of mental illness, levels of mental wellbeing usually fall into the "languishing" or "moderate" categories, with only a minority in the "flourishing" category [1-4, 40]; thus, there is considerable scope for improvement. This is important not only because wellbeing is valuable in itself, but also because wellbeing predicts a range of other positive outcomes. Numerous studies have indicated that mental wellbeing promotes workplace productivity [41, 42], cognitive performance [43, 44], social functioning [42-44], and physical health and longevity [42-47]. Furthermore, high levels of current wellbeing can predict a lower incidence of depression up to 10 years later, even after controlling for prior depression levels [48], which illustrates the modest albeit significant shared variance between wellbeing and illness. Positive emotion and subjective wellbeing can also promote mental resilience to stress and trauma [42]. Fortunately, RCTs of mental wellbeing programs [7] and positive psychology interventions [49] have shown benefits in both clinical populations and healthy participants from the general population, with outcomes including not only higher mental wellbeing but also greater quality of life, higher levels of character strengths, and lower levels of depression, anxiety, and stress [49]. These studies involved many of the positive techniques and strategies that we have included in our newly developed wellbeing app.

ReNeuWell was invented by the present authors alone, with the software programming and graphic design implemented by the digital development studio Miroma Project Factory. Here we describe the protocol for a pre-registered RCT of ReNeuWell. We hypothesize that

For peer review only - http://bmjopen.bmj.com/site/about/guidelines.xhtml

the app will produce significant improvements in mental wellbeing and reductions in psychological distress, relative to an active control version of the app.

Method

Study design

This protocol is for a single-blinded, active-controlled RCT with two conditions (training versus active control) and three measurement occasions (surveys in week 1, week 6, and week 12). The 6- and 12-week endpoints were selected because we have previously found significant effects from a smaller set of similar activities after 6 weeks [31].

Materials and procedure

The ReNeuWell app

ReNeuWell will be commercially available on the Apple store to the general public. After downloading the app and opening it for the first time, users will receive the option to continue as an ordinary paying user or to enrol in the RCT and receive free access for the 12-week study period. Thus, everyone who downloads ReNeuWell will be invited to participate. Those who select the paid version will proceed to the full version of the app and will not be included as participants. Those who select the RCT option will proceed to the Participant Information Statement and Consent Form (see Supplementary Materials), followed by screening questions to confirm eligibility. If they provide consent and pass the screening questions, participants will be randomised automatically by the app (via alternating allocation with a 1:1 ratio) to either the training condition (providing the full version of the app) or the active control condition (providing a dummy version of the app). Following randomisation, users will create an account and proceed to the app proper.

ReNeuWell comprises two main components: a recurring survey and a suite of

For peer review only

For peer review only - http://bmjopen.bmj.com/site/about/guidelines.xhtml

activities. The survey constitutes the COMPAS-W scale [5], which measures the user's wellbeing along six dimensions: Composure (dealing effectively with stress or adversity), Own-worth (a sense of autonomy and self-respect), Mastery (self-confidence, perceived control over one's environment), Positivity (happiness, optimism), Achievement (setting and pursuing goals), and Satisfaction (with one's life, health, and relationships). Upon first using the app, and every six weeks thereafter, participants complete the COMPAS-W, and their scores on the six dimensions are used by the app to generate a personalised program of daily activities for the next six weeks. Users are not shown their exact numeric scores on the COMPAS-W, but rather a screen titled *Your Results*, which provides both visual and written feedback. The written feedback comes in one of three forms, corresponding to the three categories of mental health described by Keyes [1, 2] and established for the COMPAS-W [5]: *languishing, moderate wellbeing*, and *flourishing*. Participants in the RCT will also complete additional measures within the recurring surveys. However, those in the active control condition will not be shown the *Your Results* screen.

After viewing their COMPAS-W results, participants in the training condition will be informed that the app will now generate their personalized program of activities, which constitutes the second main component of ReNeuWell. The app currently offers 18 activities, some of which come in multiple versions to cover different aspects of the relevant topic. Depending on the study outcomes, future updates to the app may add new activities or variations of existing ones. Table 1 lists each activity, along with citations of peer-reviewed studies that informed its development or support its inclusion in the app. In contrast, the active control participants will not be told that they will receive a personalised program, because the reduced app contains only a single program with three activities, designed to be neutral with regard to mental wellbeing (i.e., harmless but not beneficial). These three activities are named Acts of Novelty, Life Reminiscence, and Self Esteem, and are based on

For peer review only - http://bmjopen.bmj.com/site/about/guidelines.xhtml

the active control conditions implemented and tested in a RCT of an online wellbeing intervention [31].

Once a participant has received their program, every time they open the app (for the next six weeks), they will be taken to the *dashboard* (the "hub" screen of the app), from which they can browse their schedule of activities and select one or more to complete each day. By default, the app generates a reminder notification for users who have not opened the app for more than 72 hours, reminding them to use the app and suggesting that they try another activity. ReNeuWell also promotes engagement by allowing the user to link the app with their iPhone calendar, to record events or reminders related to their wellbeing activities. For example, for the "positive event scheduling" activity, the user can add positive events to their calendar directly from the app.

The dashboard also provides access to additional resources and features, including psychoeducational materials on mental wellbeing (with the active control app containing fewer psychoeducational resources). All users can also access contact information for mental health support services (e.g., 24-hour crisis hotlines), report an issue (e.g., a software glitch), or withdraw from the RCT via the dashboard. Participants can withdraw at any time without penalty and without having to provide a reason. Those who withdraw will have the option of continuing as a paying user. The contact information for mental health support services is also provided in the Participant Information Statement provided to all prospective participants during the informed consent process.

INSERT TABLE 1 ABOUT HERE

Instructions and timeline

Participants in both conditions will be instructed to use ReNeuWell for at least 10

For peer review only - http://bmjopen.bmj.com/site/about/guidelines.xhtml

minutes per day for the first six weeks of the 12-week study period. After week 6, participants are instructed to use the app as they see fit and informed that they will receive their final survey in week 12. For this reason, week 6 is the primary endpoint of the study, with week 12 as the follow-up. The study period is similar to those of previous RCTs of mental health apps, which have shown benefits within durations ranging from 7 days to 16 weeks [18-20, 22, 23, 26, 27]. Furthermore, it is especially important for app-based interventions to produce effects within relatively short timeframes, because if the user does experience subjective benefits soon after downloading an app, they may delete it and try one of the many alternatives.

In both conditions, each participant's schedule of daily activities is organised into weeks, with participants able to see the current week of their six-week program and any previous weeks if applicable. Participants can navigate to earlier weeks if they wish to complete an activity that they skipped, but they cannot access future activities ahead of schedule. In order for the next week of the program to be unlocked, the user must open the app at least once during the current week. Therefore, although our RCT is designed as a 12-week study, it will be possible for participants to take longer than 12 weeks to complete their programs. However, the app automatically records when a given activity or survey has been completed, allowing us to control statistically for any divergences from the intended 12-week schedule. After completing the week 12 survey and activities, training participants will be offered an additional four weeks of free access to the app (the full version this time) while control participants will be offered 12 weeks of free access to the paid version. Thus, control participants will not know that they received a reduced version of the app until they unlock the full version after week 12. Indeed, the Participant Information Statement informs prospective participants that they will be randomly allocated to one of two versions of the app, but they are not told that one of the versions is a reduced version and will not be told

For peer review only - http://bmjopen.bmj.com/site/about/guidelines.xhtml

which version they received. Each participant's app simply appears to them under the name "ReNeuWell." The study timeline is illustrated in Figure 1.

INSERT FIGURE 1 ABOUT HERE

Participants

Recruitment

The participants will be recruited from the general population of iPhone users who choose to download ReNeuWell from the Apple store. The RCT will not happen within a fixed calendar period; rather, prospective participants will be invited to participate in an ongoing manner, whenever they first open the app. All aspects of each condition (such as instructions, activities, and educational materials) will be delivered purely via the app. Therefore, participants will not need to interact with another app, a website, or any other entity (including the researchers) at any stage of the study. On the ANZCTR registry website, the anticipated date of first participant enrolment is listed as 1 February 2025. Recruitment will continue until the target sample size has been reached.

Sample size

The minimum sample size was calculated with the software package G*Power (ver. 3.1.9.2) [50, 51]. The type-1 error rate was set at the conventional level of 0.05. With the desired power of 95% and assuming a correlation of 0.5 between the repeated outcome measures, our calculations determined that a total sample size of 324 participants would be needed to detect a small group-by-time interaction effect (partial $\eta^2 = 0.01$) with two conditions and two measurement occasions (our study has three measurement occasions, but the second occasion is the primary endpoint of the study, and a power calculation with two

For peer review only - http://bmjopen.bmj.com/site/about/guidelines.xhtml

occasions is stricter than one with three). However, given that app-based interventions demand relatively low commitment from participants, it is reasonable to assume an attrition rate of at least 50% by the second measurement occasion. Indeed, a recent meta-analysis of RCTs of smartphone interventions [52] found attrition rates of 53.4% between weeks 9 and 12. Therefore, we set our target sample size at 486 participants, with 243 in each condition.

Inclusion criteria

Prospective participants will be informed that participation in the RCT is open to those who are willing to use the app for at least 10 minutes per day for at least the first 6 weeks of the 12-week study period (and up to 12 weeks), and willing to complete a 10-minute survey in weeks 1, 6, and 12. Participants will also need to meet these additional eligibility criteria:

- Aged 18 years or older (for informed consent),
- Able to understand written English (ReNeuWell is currently available in English only),
- Absence of moderate-to-severe symptoms of mental illness, as measured by a single-item question and scores on the K10 screening questionnaire [53],
- Residing in one of the following countries: Australia, New Zealand, United Kingdom, Ireland, United States, Canada (English-speaking countries),
- Possesses an iPhone (or iPod Touch) with operating system iOS 14.0 or later (although we intend to release the app on Android devices in the future, pending funding).

When ReNeuWell is first released on the Apple store, participation in the RCT will be an option only for users residing in Australia. We have approval to recruit participants in all six of the aforementioned countries, but we have decided to confine the study to Australia

For peer review only - http://bmjopen.bmj.com/site/about/guidelines.xhtml

initially, so that we will be well-positioned (being within our home country) to address any unexpected issues with the app or the trial. Barring any major technical issues or adverse events in this initial period, participation will be extended to the other five countries (and further countries pending future developments).

Screening questions

The screening questions ask for the user's age, whether they understand written English, which country they reside in, and whether (yes/no) they currently suffer from a psychological or psychiatric condition. In addition, the screening questions include the Kessler Psychological Distress Scale (K10) [53]. K10 scores of 25 or higher are considered indicative of moderate-to-severe distress and therefore of the respondent being ineligible for study participation. Those who do not pass the screening questions will be redirected to a screen whereby they can continue using the app as a paying user, if they wish. The screening questions (except the K10) are provided in the Supplementary Materials.

Survey measures

At each measurement occasion (i.e., when the participant first opens the app in week 1, week 6, and week 12), the participants will complete a 10-minute survey and will not be able to access further activities until the survey is complete. Each survey comprises a range of questionnaires, but not all measures will be administered at each measurement occasion. Table 2 outlines the questionnaire timings.

INSERT TABLE 2 ABOUT HERE

Kessler Psychological Distress Scale (K10)

For peer review only - http://bmjopen.bmj.com/site/about/guidelines.xhtml

The Kessler Psychological Distress Scale (K10) [53] will be administered during the initial screening process (as already described) and also at the beginning of the second and third surveys. A moderate-to-high K10 score at the screening stage will make the user ineligible for further participation, with K10 scores again assessed at the second and third survey time-points. The K10 comprises 10 questions on various symptoms of psychological distress (e.g., depression and anxiety symptoms), with a 5-point response scale ranging from *none of the time* to *all of the time*. Respondents are instructed to answer the questions in terms of how they have been feeling *over the past 30 days*. The K10 has exhibited acceptable reliability and validity in previous studies, and is one of the most frequently used measures in mental health research and practice [54-56].

Demographics, Health, and Lifestyle questionnaires

The Demographics questionnaire will ask about characteristics including marital status, highest education, and current occupation. The Health questionnaire will ask about the user's psychiatric history, including whether they have been diagnosed with and treated for a mental illness in the past. The Lifestyle questionnaire will ask about the user's consumption of caffeine, cigarettes, and alcohol, as well as their diet, exercise, and sleep habits. The Demographics, Health, and Lifestyle questionnaires are provided in the Supplementary Materials.

COMPAS-W Scale

The COMPAS-W Scale [5] is a 26-item measure of overall mental wellbeing and the six subcomponents of Composure, Own-worth, Mastery, Positivity, Achievement, and Satisfaction. Each item is a statement with a 5-point response scale ranging from *strongly disagree* to *strongly agree*. The respondents are instructed to answer each statement in terms

For peer review only - http://bmjopen.bmj.com/site/about/guidelines.xhtml

of how they feel *most of the time* for the week 1 survey, and then how they felt *over the past month* for later time-points. The COMPAS-W Scale has exhibited acceptable reliability and validity in several previous studies [5, 34-37, 57].

Depression, Anxiety, and Stress Scales – 21-Item Version

The Depression, Anxiety, and Stress Scales – 21-item version (DASS-21) [58, 59] is a measure of psychological distress (i.e., symptoms of depression, anxiety, and stress) comprising 21 statements with a 4-point response scale ranging from *did not apply to me at all* to *applied to me very much or most of the time*. The respondents are instructed to answer each statement in terms of how much it has applied to them *over the past week*. The DASS-21 has exhibited acceptable reliability and validity in prior non-clinical research [60, 61].

Health and Work Performance Questionnaire Scales (Employee Version)

Two sections from the employee version of the World Health Organization Health and Work Performance Questionnaire [62, 63] – scale B9 and scale B12 – will be used to measure participants' work performance. Scale B9 comprises five items asking how many days in the past four weeks the respondent missed an entire workday or part of a workday (for health or other reasons), or performed work outside their normal working hours. Scale B12 contains seven items assessing the respondent's appraisal of their own work performance in the past 4 weeks, with a 5-point response scale ranging from *none of the time* to *all of the time*.

App Satisfaction Survey

In the week 6 survey, participants will receive the ReNeuWell App Satisfaction Survey, containing a range of questions regarding the participant's opinion of the app and what they

For peer review only - http://bmjopen.bmj.com/site/about/guidelines.xhtml

liked or disliked about it. This questionnaire is provided in the Supplementary Materials.

Adverse Event Assessment

Each survey will conclude with an Adverse Event Assessment, constituting a text box in which the participant can report any aspect of the app or survey that caused discomfort or distress. If the participant has no adverse events to report, they will leave the box empty and finish the survey. This section of the survey also provides the following reminder: "In the App Settings menu, we have provided contact details for Mental Health Support Services for your country, should you ever feel distressed and require additional mental health support." The Adverse Event Assessment is provided in the Supplementary Materials.

App usage metrics

Data regarding participant usage metrics (e.g., which activities they completed, how much time they spent using the app) will be recorded within the app and via Google back-end software (Firebase).

Anonymity and confidentiality

All data collected by ReNeuWell will be automatically uploaded to a secure, dedicated server (located in Australia). On this server, users' account data (i.e., names and email addresses) are stored separately from their survey data and are only accessible using a permissions-based system. This ensures security of the data over and above encryption. The survey data will not include any personally identifying information but rather a trial participant ID number that is not shared with the user. Only the research team and app development team (Miroma Project Factory) will have access to the server. The deidentified data will then be downloaded directly from this server onto the secure internal server at

For peer review only - http://bmjopen.bmj.com/site/about/guidelines.xhtml

Neuroscience Research Australia (NeuRA) for storage and analysis. Only the research team will have access to the data.

Data availability

Per ethical requirements and participant consent, data will not be shared beyond the research team.

Statistical analysis

The primary outcomes for this RCT will be the participants' levels of both mental wellbeing (as measured by the COMPAS-W) and psychological distress (as measured by the DASS-21). The secondary outcomes will be psychological distress as measured by the K10 and work performance. Potential moderator variables include levels of usage and enjoyment of the app, past mental illness, lifestyle habits, baseline mental health levels, and demographic characteristics such as age, sex, education, or occupation.

The initial analyses will be conducted on an intention-to-treat basis, and followed by per-protocol analyses. Missing values will be estimated via multiple imputation. The hypotheses will be tested via a linear mixed model estimating the group-by-time interaction effects on the outcome variables. It is hypothesized that levels of mental wellbeing (COMPAS-W) will increase and levels of psychological distress (DASS-21) will decrease in the training condition relative to the control condition. We will also test for group-by-time interaction effects on work performance and the K10 measure of psychological distress. Any interaction effect may itself be moderated by variables such as age, sex, education, occupation, psychiatric history, lifestyle habits, app usage metrics, or baseline mental health levels. Furthermore, we will assess user engagement and retention by computing statistics on the number of participants, the levels of app usage, and the levels of attrition relative to the

For peer review only - http://bmjopen.bmj.com/site/about/guidelines.xhtml

overall number of app downloads. We will also use week 1 and week 6 survey responses to examine possible differences between participants who withdraw after these time-points and those who remain in the study until the week 12 survey, allowing us to interpret our findings with respect to any such differences.

Ethics and dissemination

This protocol has been approved by the Human Research Ethics Committee of the University of New South Wales (reference number: HC210302) and pre-registered with the Australian New Zealand Clinical Trials Registry (registration approved on 4 August 2021; trial ID number: ACTRN12621001014842p). Our findings will be published in accordance with this protocol, both in the peer-reviewed literature and on the registry website.

Public involvement

Because ReNeuWell is designed for use by the general adult population, it was appropriate to conduct several rounds of consultations with members of the general public throughout the app's development. During initial design stages, we conducted user acceptance testing (UAT) with volunteers from the general public, who provided feedback on the app's design, colours, contents, and features. The app was subsequently refined over numerous iterations, after which beta testing was conducted with local community members for feedback on usability and acceptability of the app, including identification of any bugs or feature issues. The app was then refined further and retested in-house across multiple iterations, before being "soft launched" on the Apple store for additional testing by a new group of local community members (i.e., volunteers could download ReNeuWell from the Apple store for testing purposes).

For peer review only - http://bmjopen.bmj.com/site/about/guidelines.xhtml

Discussion

The field of app-based interventions to improve mental wellbeing has yielded promising findings to date, but there remains a *research-to-retail gap* [13, 14], where many studies pertain to apps that are not publicly available, have not been tested in the general population, and/or contain proprietary wellbeing measures that have not been validated in the peer-reviewed literature. Furthermore, few previous studies have been conducted in an ecologically valid setting, where a user freely chooses to download a publicly available app from a mainstream app store and then uses the app as a standalone program without ever needing to interact with another person or entity. On the market side of the gap, many popular apps have remained available on mainstream app stores for years without ever being submitted to peer-reviewed empirical assessment, and some of these apps have product descriptions that make claims of efficacy despite the lack of empirical evidence [10, 12, 15, 33].

Alongside the research-to-retail gap, another limitation is that most wellbeing apps cover only one or a few techniques [32, 33]. The literature suggests that mindfulness exercises, which are among the techniques most commonly offered by wellbeing apps [14, 15], can indeed be effective for increasing wellbeing [7], but multi-component interventions (comprising a range of positive psychology exercises) also have efficacy [7] but have not been offered by many apps previously [32]. Furthermore, although some wellbeing apps offer evidence-based exercises [32], few if any are informed by a comprehensive, empirically validated model of wellbeing. The COMPAS-W framework [5] provides not only a multi-dimensional description of total wellbeing, but also a validated scale to measure each dimension and overall wellbeing.

ReNeuWell is a newly developed iPhone app, grounded in the COMPAS-W model of mental wellbeing [5], which addresses these openings in the literature and in the marketplace,

For peer review only - http://bmjopen.bmj.com/site/about/guidelines.xhtml

and its evaluation in our upcoming RCT will hopefully address some of the key gaps in our knowledge of mental wellbeing apps and their effectiveness. ReNeuWell provides a tailored program of daily activities and other resources designed to increase wellbeing in one or more of the six COMPAS-W dimensions. We aim to determine whether this publicly available, standalone app can promote greater wellbeing and reduce distress in the general adult population of Apple store customers who choose to use it.

For peer review only

For peer review only - http://bmjopen.bmj.com/site/about/guidelines.xhtml

Acknowledgements

The app was developed by Miroma Project Factory in consultation with the authors.

Funding statement

Costs to build the app and run this project were funded by a Mindgardens Neuroscience Grant awarded to JMG. LAE was supported by a Mindgardens Neuroscience Grant awarded to JMG, and a National Health and Medical Research Council (NHMRC) Project Grant (1122816) awarded to JMG. JMG was supported by a National Health and Medical Research Council (NHMRC) Project Grant (1122816). The study funders had no role in designing the present protocol, nor will they have any role in data collection, management, analysis, interpretation, or dissemination.

Conflicts of interest

LAE, JMG, NeuRA and UNSW NSi will receive proportional payments from any net income received from paying users of the ReNeuWell app. The trial has been pre-registered with ANZCTR and all trial outcomes will be reported in accordance with the published protocol. All trial outcomes will be made publicly available, including null results, which will be used to improve the app for future use and potential re-trials, pending funding availability.

Author contributions

LAE conducted the literature review, drafted the initial paper and subsequent versions, and contributed to the original copy of the app. JMG conceptualised the original ReNeuWell app and its design (including copy content), formulated the clinical trial study design and aims of the paper, and helped edit the original draft and revised versions. Both authors revised and edited the final version of the paper. JMG is the guarantor.

For peer review only - http://bmjopen.bmj.com/site/about/guidelines.xhtml
For peer review only

Table 1. List of activities offered in the full version of ReNeuWell, along with citations of peer-reviewed studies that informed their development or support their inclusion in the app.

**Title/Topic**	**Citations**
Acts of kindness	[31, 64-67]
Assertiveness training	[68-72]
Body language	[73-76]
Building strengths	[6, 77-79]
Colouring mandalas	[80-83]
Controlled breathing	[84-88]
Facial expressions	[89-92]
Gratitude diary	[6, 77, 93-97]
Guided meditation	[86, 98-103]
Mindfulness	[98, 101, 104-110]
Personal values	[111-114]
Positive event scheduling	[115-121]
Positive reminiscence	[31, 122-126]
Recent achievements	[127-131]
Self-compassion	[31, 132-137]
Setting goals	[138-144]
Showing empathy	[65, 145-149]
Stress and coping	[150-154]

For peer review only

For peer review only - http://bmjopen.bmj.com/site/about/guidelines.xhtml

Table 2. Questionnaires delivered at each time-point.

	Screening (Start of week 1)	Survey 1 (Start of week 1)	Survey 2 (Start of week 6)	Survey 3 (Start of week 12)
Screening questions	✓			
K10[a]	✓		✓	✓
Demographics		✓		
Health		✓		
Lifestyle		✓		
COMPAS-W[b]		✓	✓	✓
DASS-21[c]		✓	✓	✓
HPQ[d]		✓	✓	✓
App satisfaction			✓	
Adverse event assessment		✓	✓	✓

Notes: [a]K10: Kessler Psychological Distress Scale – 10-item version. [b]COMPAS-W: Composure, Own-worth, Mastery, Positivity, Achievement, and Satisfaction for Wellbeing Scale. [c]DASS-21: Depression, Anxiety, and Stress Scales – 21-item version. [d]HPQ: Health and Work Performance Questionnaire.

For peer review only

For peer review only - http://bmjopen.bmj.com/site/about/guidelines.xhtml

REFERENCES

1. Keyes CLM. The mental health continuum: From languishing to flourishing in life. Journal of Health and Social Behavior. 2002;43(2):207-22.
2. Keyes CLM. Mental illness and/or mental health? Investigating axioms of the complete state model of health. J Consult Clin Psychol. 2005;73(3):539-48.
3. Routledge KM, Burton KL, Williams LM, Harris A, Schofield PR, Clark CR, et al. Shared versus distinct genetic contributions of mental wellbeing with depression and anxiety symptoms in healthy twins. Psychiatry Res. 2016;244:65-70.
4. Mason Stephens J, Iasiello M, Ali K, van Agteren J, Fassnacht DB. The Importance of Measuring Mental Wellbeing in the Context of Psychological Distress: Using a Theoretical Framework to Test the Dual-Continua Model of Mental Health. Behav Sci. 2023;13(5):436.
5. Gatt JM, Burton KL, Schofield PR, Bryant RA, Williams LM. The heritability of mental health and wellbeing defined using COMPAS-W, a new composite measure of wellbeing. Psychiatry Res. 2014;219(1):204-13.
6. Seligman ME, Steen TA, Park N, Peterson C. Positive psychology progress: empirical validation of interventions. Am Psychol. 2005;60(5):410-21.
7. van Agteren J, Iasiello M, Lo L, Bartholomaeus J, Kopsaftis Z, Carey M, et al. A systematic review and meta-analysis of psychological interventions to improve mental wellbeing. Nat Hum Behav. 2021;5:631-52.
8. Fairburn CG, Patel V. The impact of digital technology on psychological treatments and their dissemination. Behav Res Ther. 2017;88:19-25.
9. Torous J, Staples P, Onnela JP. Realizing the potential of mobile mental health: new methods for new data in psychiatry. Curr Psychiatry Rep. 2015;17:61.
10. Badesha K, Wilde S, Dawson DL. Mental health mobile app use to manage psychological difficulties: an umbrella review. Mental Health Review Journal. 2022;27(3):241-80.
11. Linardon J, Cuijpers P, Carlbring P, Messer M, Fuller-Tyszkiewicz M. The efficacy of app-supported smartphone interventions for mental health problems: a meta-analysis of randomized controlled trials. World Psychiatry. 2019;18(3):325-36.
12. Leech T, Dorstyn D, Taylor A, Li W. Mental health apps for adolescents and young adults: A systematic review of randomised controlled trials. Children and Youth Services Review. 2021;127:106073.
13. Wasil AR, Venturo-Conerly KE, Shingleton RM, Weisz JR. A review of popular smartphone apps for depression and anxiety: Assessing the inclusion of evidence-based content. Behav Res Ther. 2019;123:103498.
14. Wasil AR, Palermo EH, Lorenzo-Luaces L, DeRubeis RJ. Is there an app for that? A review of popular apps for depression, anxiety, and well-being. Cognitive and Behavioral Practice. 2022;29:883-901.
15. Lagan S, D'Mello R, Vaidyam A, Bilden R, Torous J. Assessing mental health apps marketplaces with objective metrics from 29,190 data points from 278 apps. Acta Psychiatr Scand. 2021;144(2):201-10.
16. Leigh S, Flatt S. App-based psychological interventions: friend or foe? Evid Based Ment Health. 2015;18(4):97-9.
17. Torous J, Vaidyam A. Multiple uses of app instead of using multiple apps - a case for rethinking the digital health technology toolbox. Epidemiol Psychiatr Sci. 2020;29:e100.
18. Howells A, Ivtzan I, Eiroa-Orosa FJ. Putting the 'app' in happiness: A randomised controlled trial of a smartphone-based mindfulness intervention to enhance wellbeing. Journal of Happiness Studies: An Interdisciplinary Forum on Subjective Well-Being. 2016;17:163-85.

For peer review only - http://bmjopen.bmj.com/site/about/guidelines.xhtml

19. Mak WW, Tong AC, Yip SY, Lui WW, Chio FH, Chan AT, et al. Efficacy and Moderation of Mobile App-Based Programs for Mindfulness-Based Training, Self-Compassion Training, and Cognitive Behavioral Psychoeducation on Mental Health: Randomized Controlled Noninferiority Trial. JMIR Ment Health. 2018;5(4):e60.
20. Bakker D, Kazantzis N, Rickwood D, Rickard N. A randomized controlled trial of three smartphone apps for enhancing public mental health. Behav Res Ther. 2018;109:75-83.
21. Ehrlich C, Hennelly SE, Wilde N, Lennon O, Beck A, Messenger H, et al. Evaluation of an artificial intelligence enhanced application for student wellbeing: Pilot randomised trial of the Mind Tutor. International Journal of Applied Positive Psychology. 2023;9:435-54.
22. Gnanapragasam SN, Tinch-Taylor R, Scott HR, Hegarty S, Souliou E, Bhundia R, et al. Multicentre, England-wide randomised controlled trial of the 'Foundations' smartphone application in improving mental health and well-being in a healthcare worker population. Br J Psychiatry. 2023;222(2):58-66.
23. Golec de Zavala A, Keenan O, Ziegler M, Ciesielski P, Wahl JE, Mazurkiewicz M. App-based mindfulness training supported eudaimonic wellbeing during the COVID19 pandemic. Appl Psychol Health Well Being. 2024;16(1):42-59.
24. MacIsaac A, Mushquash AR, Wekerle C. Writing yourself well: Dispositional self-reflection moderates the effect of a smartphone app-based journaling intervention on psychological wellbeing across time. Behaviour Change. 2023;40:297-313.
25. Yuan NP, Brooks AJ, Burke MK, Crocker R, Stoner GM, Cook P, et al. My Wellness Coach: evaluation of a mobile app designed to promote integrative health among underserved populations. Transl Behav Med. 2022;12(6):752-60.
26. van Agteren J, Bartholomaeus J, Steains E, Lo L, Gerace A. Using a technology-based meaning and purpose intervention to improve well-being: A randomised controlled study. Journal of Happiness Studies: An Interdisciplinary Forum on Subjective Well-Being. 2021;22:3571-91.
27. Economides M, Martman J, Bell MJ, Sanderson B. Improvements in Stress, Affect, and Irritability Following Brief Use of a Mindfulness-based Smartphone App: A Randomized Controlled Trial. Mindfulness. 2018;9:1584-93.
28. May AD, Maurin E. Calm: A review of the mindful meditation app for use in clinical practice. Fam Syst Health. 2021;39(2):398-400.
29. Lagan S, Aquino P, Emerson MR, Fortuna K, Walker R, Torous J. Actionable health app evaluation: translating expert frameworks into objective metrics. NPJ Digit Med. 2020;3:1-8.
30. Baumel A, Muench F, Edan S, Kane JM. Objective User Engagement With Mental Health Apps: Systematic Search and Panel-Based Usage Analysis. J Med Internet Res. 2019;21(9).
31. Chilver MR, Gatt JM. Six-Week Online Multi-component Positive Psychology Intervention Improves Subjective Wellbeing in Young Adults. J Happiness Stud. 2022;23:1267-88.
32. Lau N, O'Daffer A, Yi-Frazier JP, Rosenberg AR. Popular Evidence-Based Commercial Mental Health Apps: Analysis of Engagement, Functionality, Aesthetics, and Information Quality. JMIR Mhealth Uhealth. 2021;9(7):e29689.
33. Marshall JM, Dunstan DA, Bartik W. Smartphone psychology: New approaches towards safe and efficacious mobile mental health apps. Professional Psychology: Research and Practice. 2020;51(3):214-22.
34. Chilver MR, Keller AS, Park HRP, Jamshidi J, Montalto A, Schofield PR, et al. Electroencephalography profiles as a biomarker of wellbeing: A twin study. J Psychiatr Res. 2020;126:114-21.
35. Gatt JM, Burton KLO, Routledge KM, Grasby KL, Korgaonkar MS, Grieve SM, et al.

For peer review only - http://bmjopen.bmj.com/site/about/guidelines.xhtml

A negative association between brainstem pontine grey-matter volume, well-being and resilience in healthy twins. J Psychiatry Neurosci. 2018;43(6):386-95.
36. Jamshidi J, Williams LM, Schofield PR, Park HRP, Montalto A, Chilver MR, et al. Diverse phenotypic measurements of wellbeing: Heritability, temporal stability and the variance explained by polygenic scores. Genes Brain Behav. 2020;19(8):e12694.
37. Park HRP, Quide Y, Schofield PR, Williams LM, Gatt JM. Grey matter covariation and the role of emotion reappraisal in mental wellbeing and resilience after early life stress exposure. Transl Psychiatry. 2022;12:85.
38. Park HRP, Chilver MR, Montalto A, Jamshidi J, Schofield PR, Williams LM, et al. Associations between mental wellbeing and fMRI neural bases underlying responses to positive emotion in a twin sample. Psychol Med. 2023;53(4):1215-23.
39. Routledge KM, Burton KLO, Williams LM, Harris A, Schofield PR, Clark CR, et al. The shared and unique genetic relationship between mental well-being, depression and anxiety symptoms and cognitive function in healthy twins. Cogn Emot. 2017;31(7):1465-79.
40. Huppert FA, So TT. Flourishing Across Europe: Application of a New Conceptual Framework for Defining Well-Being. Soc Indic Res. 2013;110:837-61.
41. Walsh LC, Boehm JK, Lyubomirsky S. Does happiness promote career success? Revisiting the evidence. Journal of Career Assessment. 2018;26(2):199-219.
42. Egan LA, Park HRP, Lam J, Gatt JM. Resilience to Stress and Adversity: A Narrative Review of the Role of Positive Affect. Psychol Res Behav Manag. 2024;17:2011-38.
43. Huppert FA. Psychological well-being: evidence regarding its causes and consequences. Appl Psychol Health Well Being. 2009;1(2):137-64.
44. Lyubomirsky S, King L, Diener E. The benefits of frequent positive affect: Does happiness lead to success? Psychol Bull. 2005;131(6):803-55.
45. Fredrickson BL. The value of positive emotions. American Scientist. 2003;91:330-5.
46. Kubzansky LD, Kim ES, Boehm JK, Davidson RJ, Huffman JC, Loucks EB, et al. Interventions to Modify Psychological Well-Being: Progress, Promises, and an Agenda for Future Research. Affect Sci. 2023;4:174-84.
47. VanderWeele TJ, Chen Y, Long K, Kim ES, Trudel-Fitzgerald C, Kubzansky LD. Positive Epidemiology? Epidemiology. 2020;31(2):189-93.
48. Wood AM, Joseph S. The absence of positive psychological (eudemonic) well-being as a risk factor for depression: a ten year cohort study. J Affect Disord. 2010;122(3):213-7.
49. Carr A, Cullen K, Keeney C, Canning C, Mooney O, Chinseallaigh E, et al. Effectiveness of positive psychology interventions: a systematic review and meta-analysis. The Journal of Positive Psychology. 2021;16(6):749-69.
50. Faul F, Erdfelder E, Lang AG, Buchner A. G*Power 3: a flexible statistical power analysis program for the social, behavioral, and biomedical sciences. Behav Res Methods. 2007;39(2):175-91.
51. Faul F, Erdfelder E, Buchner A, Lang AG. Statistical power analyses using G*Power 3.1: tests for correlation and regression analyses. Behav Res Methods. 2009;41(4):1149-60.
52. Linardon J, Fuller-Tyszkiewicz M. Attrition and adherence in smartphone-delivered interventions for mental health problems: A systematic and meta-analytic review. J Consult Clin Psychol. 2020;88(1):1-13.
53. Kessler RC, Andrews G, Colpe LJ, Hiripi E, Mroczek DK, Normand SL, et al. Short screening scales to monitor population prevalences and trends in non-specific psychological distress. Psychol Med. 2002;32:959-76.
54. Brooks RT, Beard J, Steel Z. Factor structure and interpretation of the K10. Psychol Assess. 2006;18(1):62-70.
55. Merson F, Newby J, Shires A, Millard M, Mahoney A. The temporal stability of the Kessler Psychological Distress Scale. Australian Psychologist. 2021;56(1):38-45.

For peer review only - http://bmjopen.bmj.com/site/about/guidelines.xhtml

56. Slade T, Grove R, Burgess P. Kessler Psychological Distress Scale: normative data from the 2007 Australian National Survey of Mental Health and Wellbeing. Australian and New Zealand Journal of Psychiatry. 2011;45(4):308-16.
57. Gatt JM, Alexander R, Emond A, Foster K, Hadfield K, Mason-Jones A, et al. Trauma, Resilience, and Mental Health in Migrant and Non-Migrant Youth: An International Cross-Sectional Study Across Six Countries. Front Psychiatry. 2020;10:997.
58. Lovibond SH, Lovibond PF. Manual for the Depression Anxiety Stress Scales. 2nd edition ed. Sydney: Psychology Foundation of Australia; 1995.
59. Lovibond PF, Lovibond SH. The structure of negative emotional states: Comparison of the Depression Anxiety Stress Scales (DASS) with the Beck Depression and Anxiety Inventories. Behaviour Research and Therapy. 1995;33(3):335-43.
60. Osman A, Wong JL, Bagge CL, Freedenthal S, Gutierrez PM, Lozano G. The Depression Anxiety Stress Scales-21 (DASS-21): further examination of dimensions, scale reliability, and correlates. J Clin Psychol. 2012;68(12):1322-38.
61. Zanon C, Brenner RE, Baptista MN, Vogel DL, Rubin M, Al-Darmaki FR, et al. Examining the Dimensionality, Reliability, and Invariance of the Depression, Anxiety, and Stress Scale-21 (DASS-21) Across Eight Countries. Assessment. 2021;28(6):1531-44.
62. Kessler RC, Barber C, Beck A, Berglund P, Cleary PD, McKenas D, et al. The World Health Organization Health and Work Performance Questionnaire (HPQ). J Occup Environ Med. 2003;45(2):156-74.
63. Kessler RC, Ames M, Hymel PA, Loeppke R, McKenas DK, Richling DE, et al. Using the World Health Organization Health and Work Performance Questionnaire (HPQ) to evaluate the indirect workplace costs of illness. J Occup Environ Med. 2004;46(6 Suppl):S23-S37.
64. Curry OS, Rowland LA, Van Lissa CJ, Zlotowitz S, McAlaney J, Whitehouse H. Happy to help? A systematic review and meta-analysis of the effects of performing acts of kindness on the well-being of the actor. Journal of Experimental Social Psychology. 2018;76:320-9.
65. Kirby JN. Compassion interventions: The programmes, the evidence, and implications for research and practice. Psychology and Psychotherapy: Theory, Research and Practice. 2017;90(3):432-55.
66. Laguna M, Mazur Z, Kedra M, Ostrowski K. Interventions stimulating prosocial helping behavior: A systematic review. Journal of Applied Social Psychology. 2020;50(11):676-96.
67. O'Brien E, Kassirer S. People are slow to adapt to the warm glow of giving. Psychological Science. 2019;30(2):193-204.
68. Alberti RE, Emmons ML. A manual for assertiveness trainers. New York: Impact; 1990.
69. Shaffer CS, Shapiro J, Sank LI, Coghlan DJ. Positive changes in depression, anxiety, and assertion following individual and group cognitive behavior therapy intervention. Cognitive Therapy and Research. 1981;5(2):149-57.
70. Speed BC, Goldstein BL, Goldfried MR. Assertiveness training: A forgotten evidence-based treatment. Clinical Psychology: Science and Practice. 2018;25(1):e12216.
71. Vagos P, Pereira A. A cognitive perspective for understanding and training assertiveness. European Psychologist. 2016;21(2):109-21.
72. Zappe C, Epstein D. Assertive training. Journal of Psychosocial Nursing and Mental Health Services. 1987;25(8):23-6.
73. de Gelder B, Huis In 'T Veld EMJ. Cultural differences in emotional expressions and body language. In: Chiao JY, Li S, Seligman R, Turner R, editors. The Oxford Handbook of Cultural Neuroscience. New York: Oxford University Press; 2015. p. 223–34.

For peer review only - http://bmjopen.bmj.com/site/about/guidelines.xhtml

74. Olszewski A, Panorska A, Gillam SL. Training verbal and nonverbal communication interview skills to adolescents. Communication Disorders Quarterly. 2017;38(4):206-18.
75. Peterson RT. An Examination of the Relative Effectiveness of Training in Nonverbal Communication: Personal Selling Implications. J Market Educ. 2005;27(2):143-50.
76. Smith LW, Delgado RA. Body language: The interplay between positional behavior and gestural signaling in the genus Pan and its implications for language evolution. Am J Phys Anthropol. 2015;157(4):592-602.
77. Gander F, Proyer RT, Ruch W, Wyss T. Strength-based positive interventions: Further evidence for their potential in enhancing well-being and alleviating depression. Journal of Happiness Studies: An Interdisciplinary Forum on Subjective Well-Being. 2013;14:1241-59.
78. Proyer RT, Gander F, Wellenzohn S, Ruch W. Positive psychology interventions in people aged 50-79 years: Long-term effects of placebo-controlled online interventions on well-being and depression. Aging & Mental Health. 2014;18(8):997-1005.
79. Rust T, Diessner R, Reade L. Strengths only or strengths and relative weaknesses? A preliminary study. The Journal of Psychology: Interdisciplinary and Applied. 2009;143(5):465-76.
80. Curry NA, Kasser T. Can coloring mandalas reduce anxiety? Art Therapy. 2005;22(2):81-5.
81. Holt NJ, Furbert L, Sweetingham E. Cognitive and affective benefits of coloring: Two randomized controlled crossover studies. Art Therapy. 2019;36(4):200-8.
82. Store SJ, Jakobsson N. The effect of mandala coloring on state anxiety: A systematic review and meta-analysis. Art Therapy. 2022;39(4):173-81.
83. van der Vennet R, Serice S. Can coloring mandalas reduce anxiety? A replication study. Art Therapy. 2012;29(2):87-92.
84. Hopper SI, Murray SL, Ferrara LR, Singleton JK. Effectiveness of diaphragmatic breathing for reducing physiological and psychological stress in adults: a quantitative systematic review. JBI Database System Rev Implement Rep. 2019;17(9):1855-76.
85. Jerath R, Crawford MW, Barnes VA, Harden K. Self-regulation of breathing as a primary treatment for anxiety. Appl Psychophysiol Biofeedback. 2015;40:107-15.
86. Mandlik GV, Siopis G, Nguyen B, Ding D, Edwards KM. Effect of a single session of yoga and meditation on stress reactivity: A systematic review. Stress Health. 2023;40(3):e3324.
87. Shao R, Man ISC, Lee TMC. The effect of slow-paced breathing on cardiovascular and emotion functions: A meta-analysis and systematic review. Mindfulness. 2024;15:1-18.
88. Zaccaro A, Piarulli A, Laurino M, Garbella E, Menicucci D, Neri B, et al. How breath-control can change your life: A systematic review on psycho-physiological correlates of slow breathing. Frontiers in Human Neuroscience. 2018;12:353.
89. Hinsz VB, Tomhave JA. Smile and (half) the world smiles with you, frown and you frown alone. Personality and Social Psychology Bulletin. 1991;17(5):586-92.
90. Hwang HC, Matsumoto D. Facial expressions. In: Matsumoto D, Hwang HC, Frank MG, editors. APA Handbook of Nonverbal Communication: American Psychological Association; 2016. p. 257-87.
91. Penton-Voak IS, Munafo MR, Looi CY. Biased facial-emotion perception in mental health disorders: A possible target for psychological intervention? Current Directions in Psychological Science. 2017;26(3):294-301.
92. Strnadelova B, Halamova J, Kanovsky M. Identification of Primary Facial Emotions in Relation to Level of Self-Criticism. Perception. 2019;48(10):948-67.
93. Hartanto A, Kaur M, Kasturiratna KTAS, Quek FYX, Majeed NM. A critical examination of the effectiveness of gratitude intervention on well-Being Outcomes: A within-

For peer review only - http://bmjopen.bmj.com/site/about/guidelines.xhtml

person experimental daily diary approach. J Posit Psychol. 2023;18(6):942-57.
94. Heckendorf H, Lehr D, Ebert DD, Freund H. Efficacy of an internet and app-based gratitude intervention in reducing repetitive negative thinking and mechanisms of change in the intervention's effect on anxiety and depression: Results from a randomized controlled trial. Behav Res Ther. 2019;119:103415.
95. Kloos N, Austin J, van't Klooster J-W, Drossaert C, Bohlmeijer E. Appreciating the good things in life during the covid-19 pandemic: A randomized controlled trial and evaluation of a gratitude app. Journal of Happiness Studies: An Interdisciplinary Forum on Subjective Well Being. 2022;23:4001-25.
96. Southwell S, Gould E. A randomised wait list-controlled pre-post-follow-up trial of a gratitude diary with a distressed sample. The Journal of Positive Psychology. 2017;12(6):579-93.
97. Tolcher K, Cauble M, Downs A. Evaluating the effects of gratitude interventions on college student well-being. J Am Coll Health. 2022;72(4):1321-5.
98. Gal E, Stefan S, Cristea IA. The efficacy of mindfulness meditation apps in enhancing users' well-being and mental health related outcomes: a meta-analysis of randomized controlled trials. J Affect Disord. 2021;279:131-42.
99. Luberto CM, Shinday N, Song R, Philpotts LL, Park ER, Fricchione GL, et al. A Systematic Review and Meta-analysis of the Effects of Meditation on Empathy, Compassion, and Prosocial Behaviors. Mindfulness. 2018;9:708-24.
100. Montero-Marin J, Garcia-Campayo J, Perez-Yus MC, Zabaleta-Del-Olmo E, Cuijpers P. Meditation techniques v. relaxation therapies when treating anxiety: a meta-analytic review. Psychol Med. 2019;49(13):2118-33.
101. Vancampfort D, Stubbs B, Van Damme T, Smith L, Hallgren M, Schuch F, et al. The efficacy of meditation-based mind-body interventions for mental disorders: A meta-review of 17 meta-analyses of randomized controlled trials. J Psychiatr Res. 2021;134:181-91.
102. Zeng X, Chiu CPK, Wang R, Oei TPS, Leung FYK. The effect of loving-kindness meditation on positive emotions: a meta-analytic review. Front Psychol. 2015;6:1693.
103. Zheng Y, Yan L, Chen Y, Zhang X, Sun W, Lv J, et al. Effects of loving-kindness and compassion meditation on anxiety: A systematic review and meta-analysis. Mindfulness. 2023;14:1021-37.
104. Chen B, Yang T, Xiao L, Xu C, Zhu C. Effects of Mobile Mindfulness Meditation on the Mental Health of University Students: Systematic Review and Meta-analysis. J Med Internet Res. 2023;25:e39128.
105. Goldberg SB, Riordan KM, Sun S, Davidson RJ. The empirical status of mindfulness-based interventions: A systematic review of 44 meta-analyses of randomized controlled trials. Perspectives on Psychological Science. 2022;17(1):108-30.
106. Gotink RA, Meijboom R, Vernooij MW, Smits M, Hunink MG. 8-week Mindfulness Based Stress Reduction induces brain changes similar to traditional long-term meditation practice - A systematic review. Brain Cogn. 2016;108:32-41.
107. Johnson BT, Acabchuk RL, George EA, Nardi W, Sun S, Salmoirago-Blotcher E, et al. Mental and physical health impacts of mindfulness training for college undergraduates: A systematic review and meta-analysis of randomized controlled trials. Mindfulness. 2023;14:2077-96.
108. Schwartz K, Ganster FM, Tran US. Mindfulness-Based Mobile Apps and Their Impact on Well-Being in Nonclinical Populations: Systematic Review of Randomized Controlled Trials. J Med Internet Res. 2023;25:e44638.
109. Tan ZYA, Wong SH, Cheng LJ, Lau ST. Effectiveness of mobile-based mindfulness interventions in improving mindfulness skills and psychological outcomes for adults: A systematic review and meta-regression. Mindfulness. 2022;13:2379-95.

For peer review only - http://bmjopen.bmj.com/site/about/guidelines.xhtml

110. Taylor H, Strauss C, Cavanagh K. Can a little bit of mindfulness do you good? A systematic review and meta-analyses of unguided mindfulness-based self-help interventions. Clin Psychol Rev. 2021;89:102078.
111. Gloster AT, Walder N, Levin ME, Twohig MP, Karekla M. The empirical status of acceptance and commitment therapy: A review of meta-analyses. Journal of Contextual Behavioral Science. 2020;18:181-92.
112. Serowik KL, Khan AJ, LoCurto J, Orsillo SM. The conceptualization and measurement of values: A review of the psychometric properties of measures developed to inform values work with adults. Journal of Psychopathology and Behavioral Assessment. 2018;40:615-35.
113. Stenhoff A, Steadman L, Nevitt S, Benson L, White RG. Acceptance and commitment therapy and subjective wellbeing: A systematic review and meta-analyses of randomised controlled trials in adults. Journal of Contextual Behavioral Science. 2020;18:256-72.
114. Witteman HO, Ndjaboue R, Vaisson G, Dansokho SC, Arnold B, Bridges JFP, et al. Clarifying Values: An Updated and Expanded Systematic Review and Meta-Analysis. Med Decis Making. 2021;41(7):801-20.
115. Han A, Kim TH. Effects of internet-delivered behavioral activation on individuals with depressive symptoms: A systematic review and meta-analysis. J Psychiatr Res. 2022;152:104-18.
116. Huguet A, Miller A, Kisely S, Rao S, Saadat N, McGrath PJ. A systematic review and meta-analysis on the efficacy of Internet-delivered behavioral activation. J Affect Disord. 2018;235:27-38.
117. Layous K, Chancellor J, Lyubomirsky S. Positive activities as protective factors against mental health conditions. J Abnorm Psychol. 2014;123(1):3-12.
118. Ong AD, Lee S. Variety in pleasant activities is associated with improved mental health: Evidence from two national samples of U.S. adults. Affective Science. 2023;5:90-8.
119. Panaite V, Yoon S, Devendorf AR, Kashdan TB, Goodman FR, Rottenberg J. Do positive events and emotions offset the difficulties of stressful life events? A daily diary investigation of depressed adults. Personality and Individual Differences. 2022;186(Part A):111379.
120. Spates CR, Padalino R, Hale AC, Germain CS, Nimmo K, Kohler R. A review of web-based technology in behavioural activation. Clinical Psychologist. 2016;20(1):27-35.
121. Vanderkruik R, Whisman MA. A cross-cultural examination of pleasant events and depressive symptoms. Journal of Clinical Psychology. 2021;77:1384-93.
122. Bryant FB, Smart CM, King SP. Using the past to enhance the present: Boosting happiness through positive reminiscence. Journal of Happiness Studies: An Interdisciplinary Forum on Subjective Well-Being. 2005;6:227-60.
123. Hallford DJ, Hardgrove S, Sanam M, Oliveira S, Pilon M, Duran T. Remembering for resilience: Brief cognitive-reminiscence therapy improves psychological resources and mental well-being in young adults. Applied Psychology: Health and Well Being. 2022;14:1004-21.
124. Miguel-Alvaro A, Guillen AI, Contractor AA, Crespo M. Positive memory intervention techniques: a scoping review. Memory. 2021;29(6):793-810.
125. Tam W, Poon SN, Mahendran R, Kua EH, Wu XV. The effectiveness of reminiscence-based intervention on improving psychological well-being in cognitively intact older adults: A systematic review and meta-analysis. Int J Nurs Stud. 2021;114:103847.
126. Westerhof GJ, Bohlmeijer E, Webster JD. Reminiscence and mental health: A review of recent progress in theory, research and interventions. Ageing & Society. 2010;30:697-721.
127. Dickens LR, Robins RW. Pride: A meta-analytic project. Emotion. 2022;22(5):1071-

For peer review only - http://bmjopen.bmj.com/site/about/guidelines.xhtml

87.
128. Ding K, Liu J. Comparing gratitude and pride: evidence from brain and behavior. Cogn Affect Behav Neurosci. 2022;22:1199-214.
129. Lin D, Bi J, Zhang X, Zhu F, Wang Y. Successful emotion regulation via cognitive reappraisal in authentic pride: Behavioral and event-related potential evidence. Front Hum Neurosci. 2022;16:983674.
130. Van Doren N, Tharp JA, Johnson SL, Staudenmaier PJ, Anderson C, Freeman MA. Perseverance of effort is related to lower depressive symptoms via authentic pride and perceived power. Personality and Individual Differences. 2019;137:45-9.
131. Weidman AC, Tracy JL, Elliot AJ. The benefits of following your pride: Authentic pride promotes achievement. Journal of Personality. 2016;84(5):607-22.
132. Allen AB, Leary MR. Self-compassion, stress, and coping. Social and Personality Psychology Compass. 2010;4(2):107-18.
133. Barnard LK, Curry JF. Self-compassion: Conceptualizations, correlates, & interventions. Review of General Psychology. 2011;15(4):289-303.
134. Ferrari M, Hunt C, Harrysunker A, Abbott MJ, Beath AP, Einstein DA. Self-compassion interventions and psychosocial outcomes: A meta-analysis of RCTs. Mindfulness. 2019;10:1455-73.
135. Neff KD. The development and validation of a scale to measure self-compassion. Self and Identity. 2003;2(3):223-50.
136. Neff KD, Kirkpatrick KL, Rude SS. Self-compassion and adaptive psychological functioning. Journal of Research in Personality. 2007;41(1):139-54.
137. Zessin U, Dickhauser O, Garbade S. The relationship between self-compassion and well-being: A meta-analysis. Applied Psychology: Health and Well-Being. 2015;7(3):340-64.
138. Bjorvatn K, Ekstrom M, Garcia Pires AJ. Setting goals for keystone habits improves labor market prospects and life satisfaction for unemployed youth: Experimental evidence from Norway. Journal of Economic Behavior & Organization. 2021;188:1109-23.
139. Bovend'Eerdt TJH, Botell RE, Wade DT. Writing SMART rehabilitation goals and achieving goal attainment scaling: a practical guide. Clin Rehabil. 2009;23(4):352-61.
140. Cairns AJ, Kavanagh DJ, Dark F, McPhail SM. Goal setting improves retention in youth mental health: a cross-sectional analysis. Child Adolesc Psychiatry Ment Health. 2019;13:31.
141. Carrillo A, Rubio-Aparicio M, Molinari G, Enrique A, Sanchez-Meca J, Banos RM. Effects of the Best Possible Self intervention: A systematic review and meta-analysis. PLoS One. 2019;14(9):e0222386.
142. Elzubeir K, Dye S. Can amount and duration of seclusion be reduced in psychiatric intensive care units by agreeing SMART goals with patients? Journal of Psychiatric Intensive Care. 2017;13(2):109-16.
143. Epton T, Currie S, Armitage CJ. Unique effects of setting goals on behavior change: Systematic review and meta-analysis. Journal of Consulting and Clinical Psychology. 2017;85(12):1182-98.
144. Weintraub J, Cassell D, DePatie TP. Nudging flow through 'SMART' goal setting to decrease stress, increase engagement, and increase performance at work. Journal of Occupational and Organizational Psychology. 2021;94:230-58.
145. Batt-Rawden SA, Chisolm MS, Anton B, Flickinger TE. Teaching empathy to medical students: An updated, systematic review. Academic Medicine. 2013;88(8):1171-7.
146. Bell H. Creative interventions for teaching empathy in the counseling classroom. Journal of Creativity in Mental Health. 2018;13(1):106-20.
147. D'Souza PC, Rasquinha SL, D'Souza TL, Jain A, Kulkarni V, Pai K. Effect of a single-session communication skills training on empathy in medical students. Academic

For peer review only - http://bmjopen.bmj.com/site/about/guidelines.xhtml

Psychiatry. 2020;44:289-94.
148. Davis JR, Gold GJ. An examination of emotional empathy, attributions of stability, and the link between perceived remorse and forgiveness. Personality and Individual Differences. 2011;50(3):392-7.
149. Riess H, Kelley JM, Bailey RW, Dunn EJ, Phillips M. Empathy training for resident physicians: A randomized controlled trial of a neuroscience-informed curriculum. Journal of General Internal Medicine. 2012;27(10):1280-6.
150. Carver CS. You want to measure coping but your protocol's too long: Consider the Brief COPE. International Journal of Behavioral Medicine. 1997;4(1):92-100.
151. Carver CS, Scheier MF, Weintraub JK. Assessing coping strategies: A theoretically based approach. Journal of Personality and Social Psychology. 1989;56(2):267-83.
152. Fischer R, Scheunemann J, Moritz S. Coping strategies and subjective well-being: Context matters. Journal of Happiness Studies: An Interdisciplinary Forum on Subjective Well-Being. 2021;22:3413-34.
153. Meng X, D'Arcy C. Coping strategies and distress reduction in psychological well-being? A structural equation modelling analysis using a national population sample. Epidemiology and Psychiatric Sciences. 2016;25(4):370-83.
154. Skinner EA, Edge K, Altman J, Sherwood H. Searching for the structure of coping: A review and critique of category systems for classifying ways of coping. Psychological Bulletin. 2003;129(2):216-69.

For peer review only

For peer review only - http://bmjopen.bmj.com/site/about/guidelines.xhtml

Figure 1. Study timeline.

For peer review only

For peer review only - http://bmjopen.bmj.com/site/about/guidelines.xhtml

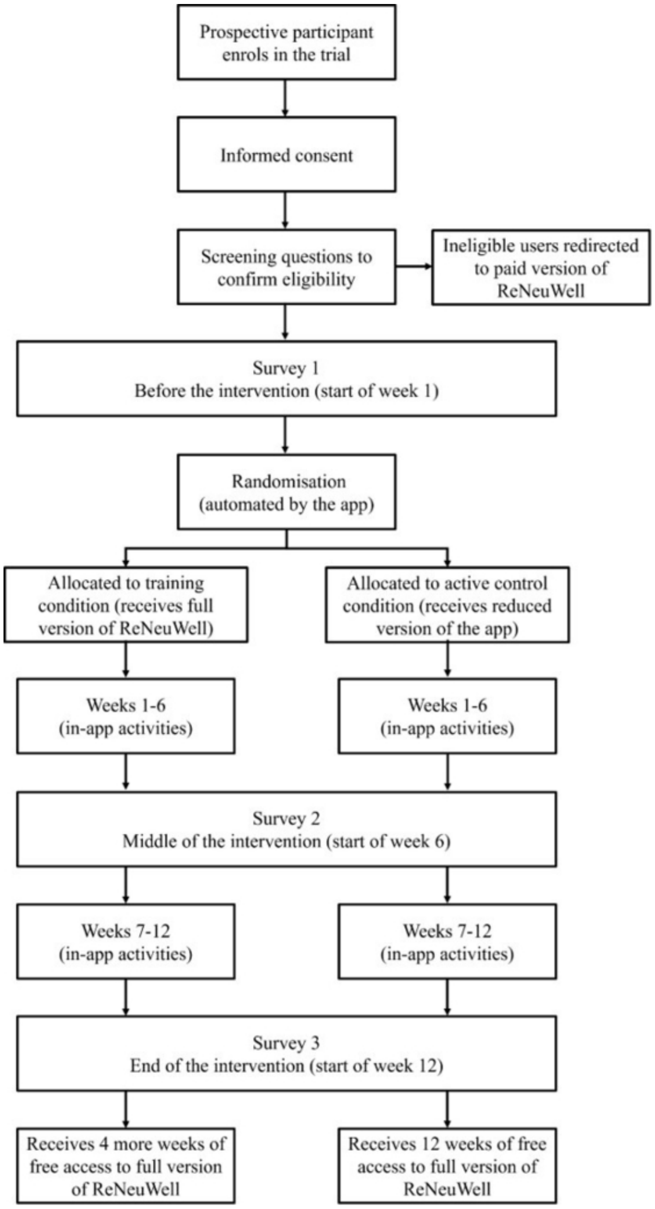

Figure 1: Study timeline.

47x87mm (300 x 300 DPI)

Supplementary Materials

for

The ReNeuWell mental wellbeing app:

Protocol for a randomised controlled trial

Luke A. Egan[1] and Justine M. Gatt[1,2,3*]

[1] Centre for Wellbeing, Resilience and Recovery, Neuroscience Research Australia, Randwick, NSW, Australia.

[2] School of Psychology, University of New South Wales, Kensington, NSW, Australia.

[3] Black Dog Institute, Randwick, NSW, Australia

***Corresponding author:** Associate Professor Justine Gatt; Phone: +612 9399 1812; Postal address: Neuroscience Research Australia, Barker St, Randwick, NSW 2031, Australia; E-mail address: j.gatt@unsw.edu.au

For peer review only - http://bmjopen.bmj.com/site/about/guidelines.xhtml

Screening questions (in addition to K10)

1. How old are you?

2. Do you understand written English?

3. What country do you currently live in?
   - Australia
   - Canada
   - Ireland
   - New Zealand
   - USA
   - UK
   - Other: ______________________

4. Do you currently suffer from a psychiatric or psychological condition? (e.g., anxiety disorder, depression, bipolar disorder, PTSD, phobia, psychosis, schizophrenia, eating disorder etc)

For peer review only - http://bmjopen.bmj.com/site/about/guidelines.xhtml

DEMOGRAPHICS

1	What is your age (in years)?	
2	Gender	• Male<br>• Female<br>• Non-binary / Other
3	What country do you currently live in?	Australia<br>Canada<br>Ireland<br>New Zealand<br>USA<br>UK
4	What state/ province/ county do you currently live in?	______________________________
5	What is your current marital status?	• Single<br>• Have a boyfriend/girlfriend<br>• Married / De facto (living with partner)<br>• Separated / Divorced<br>• Widowed
6	Height	_______ cm or _______ feet/inches
7	Weight	_______ kg or ________ pounds
8	Highest education completed	• High School<br>• Trade Certificate<br>• Graduate diploma or graduate certificate<br>• Undergraduate degree (Bachelor)<br>• Postgraduate degree (Master / PhD)
9	What is your current employment status?	• Full-time<br>• Part-time<br>• Studying and working<br>• Studying only<br>• Unemployed and looking for work<br>• Not doing paid work and not looking for work
10	What is your current main occupation?	______________________________
11	What is your role in your organization? (e.g., employee, manager, executive manager)	______________________________

12	Please think of the following 10-point scale like a ladder, representing where people stand in their society. People define society in different ways; please define it in whatever way is most meaningful to you.<br>At the top of the scale ("10") are the people who have the highest standing in their society, and at the bottom ("1") are the people who have the lowest standing in their society.<br>Where would you place yourself on this scale?	• 10. Highest standing in society<br>• 9.<br>• 8.<br>• 7.<br>• 6.<br>• 5.<br>• 4.<br>• 3.<br>• 2.<br>• 1. Lowest standing in society

HEALTH

1	Have you ever been diagnosed with a psychiatric or psychological condition? (e.g., eating disorder, anxiety disorder, depression, social phobia, PTSD)?	• Yes<br>• No<br>• Prefer not to answer
If "No" or "Prefer not to answer", finish this section.		
2	Which psychiatric disorders have you been diagnosed with? Please tick all that apply.	• Eating Disorder<br>• Generalized Anxiety Disorder<br>• Panic Disorder<br>• Post-traumatic stress disorder (PTSD)<br>• Social Phobia / Social Anxiety Disorder<br>• Major Depression<br>• Bipolar Disorder (Manic Depression)<br>• Schizophrenia<br>• Obsessive Compulsive Disorder<br>• Personality Disorder (e.g. Borderline Personality Disorder)<br>• Tourette's Disorder<br>• Other: ______________________________
3	What age were you when you were first diagnosed?	____________ years old
4	For how long did you suffer from this condition?	• Less than 1 year<br>• 1 – 3 years<br>• 3 – 5 years<br>• Ongoing

5	Did you receive treatment for this condition? Please tick all that apply.	• No treatment<br>• I received behavioural therapy (e.g., CBT): ___________ (please specify)<br>• I received medication treatment (e.g., antidepressants): ___________ (please specify)<br>• Alternative therapies (e.g., homeopathy, naturopathy): ___________ (please specify)

LIFESTYLE

1	On average, how many cups of caffeinated drinks (coffee, coke or energy drinks) do you drink per day?	• None<br>• 1 – 2 cups<br>• 3 – 4 cups<br>• Over 5 cups
2	Has there ever been a time when you smoked cigarettes regularly? That is, at least one cigarette per day for 3 months or longer?	Yes<br>No
If "No", go to question #9.		
3	How old were you when you first started smoking cigarettes regularly?	__________ years old
4	In total, how many years have you smoked cigarettes regularly?	__________ years
5	Over the time you smoked regularly, how many cigarettes on average did you smoke per day?	• 1-5 cigarettes per day<br>• 6-10 cigarettes per day<br>• 11-20 cigarettes per day<br>• 21-30 cigarettes per day<br>• More than a pack a day
6	Are you currently smoking cigarettes regularly?	Yes<br>No
If "Yes", go to question #7 then question #9.<br>If "No", go to question #8.		
7	Over the last year, how many cigarettes on average did you smoke per day?	• 1-5 cigarettes per day<br>• 6-10 cigarettes per day<br>• 11-20 cigarettes per day<br>• 21-30 cigarettes per day<br>• More than a pack a day

For peer review only - http://bmjopen.bmj.com/site/about/guidelines.xhtml

8	When did you stop smoking cigarettes regularly?	• I stopped smoking in the past 3 months<br>• I stopped smoking in the past 3-6 months<br>• I stopped smoking in the past 6-12 months<br>• I stopped smoking last year<br>• I stopped smoking when I was about: ____ years old
9	How often do you have a drink containing alcohol?	• Never<br>• Monthly or less<br>• 2-4 times a month<br>• 2-3 times a week<br>• 4 or more times a week
10	How many drinks containing alcohol do you have on a typical day when you are drinking?	• 1 or 2<br>• 3 or 4<br>• 5 or 6<br>• 7 to 9<br>• 10 or more
11	How often do you have 6 or more drinks on one occasion?	• Never<br>• Less than monthly<br>• Monthly<br>• Weekly<br>• Daily or almost daily
12	On average, how many times a week do you exercise (e.g., strength training, aerobic exercise, or activities such as running, swimming, strenuous walking or bike riding) for at least 30 minutes at a time?	• None<br>• 1 – 2 times<br>• 3 – 4 times<br>• 5 – 6 times<br>• Daily
13	On average, how many times a week do you eat fruit and vegetables?	• None<br>• 1 – 2 times<br>• 3 – 4 times<br>• 5 – 6 times<br>• Daily
14	How many hours of sleep do you usually get per night?	• Less than 3 hours<br>• 3 – 5 hours<br>• 6 – 8 hours<br>• Over 8 hours

For peer review only - http://bmjopen.bmj.com/site/about/guidelines.xhtml

APP SATISFACTION SURVEY

1. Overall, how important is it to you to improve your own wellbeing?

• Very important
• Moderately important
• Neutral
• Slightly important
• Not important

2. How many apps have you previously downloaded or used that aim to improve your wellbeing (including meditation and mindfulness apps)?

• None previously. This is my first time
• 1-2 apps previously
• 3 or more apps previously

3. Have you previously attended group classes like yoga and meditation?

• No, never
• Yes, I have attended a few classes previously
• Yes, I attend yoga and/or meditative classes on a regular basis

4. Overall, how helpful do you feel the app was in increasing your mental wellbeing (e.g., mental health, positivity, emotional health)?

• Very helpful
• Quite a lot
• Somewhat
• A little
• Not at all helpful

5. Overall, how helpful do you feel the app was in increasing your physical wellbeing (e.g., vigour, fitness, sleep)?

• Very helpful
• Quite a lot
• Somewhat
• A little
• Not at all helpful

6. When using the app, how easy was it to find core features and navigate the app?

• Very easy
• Easy
• Neutral
• Difficult
• Very difficult

7. Do you see the value in using the app?

- Yes, absolutely
- Yes, mostly
- Sometimes
- Not always
- Not at all

8. What is the likelihood you will continue using the app for your own wellbeing?

- Extremely likely
- Likely
- Neutral
- Unlikely
- Extremely unlikely

9. Do you prefer to use this app over other wellbeing apps?

- Yes
- No

If no, what other apps do you prefer and why? ______________________

10. What did you like the most about the app? ______________________

11. What activity did you like the most? ______________________

12. What did you dislike about the app? ______________________

13. What other information would you like to see in the app? ______________________

14. Other suggestions to improve the experience? ______________________

Adverse Event Assessment

Thank you for taking the time to share your answers and feedback. Your responses are anonymous and confidential, and the survey responses will be used to help develop our app further.

If any aspect of this survey or app caused you distress, please describe in the space below, including which items or features (otherwise you can leave it blank):

____
____
____
____

In the App Settings menu, we have provided contact details for Mental Health Support Services for your country, should you ever feel distressed and require additional mental health support.

For peer review only - http://bmjopen.bmj.com/site/about/guidelines.xhtml

PARTICIPANT INFORMATION STATEMENT

A randomised controlled trial of the ReNeuWell® wellbeing app

Chief Investigator: Dr Justine M. Gatt

1. What is this research study about?
The study measures people's responses to the ReNeuWell® app. You are invited to participate because you have installed the app on your phone.

2. Who is conducting this study?
The study is being run by Dr Justine M. Gatt from Neuroscience Research Australia (NeuRA) and the University of New South Wales (UNSW). The study is funded by the Mindgardens Neuroscience Network and the National Health and Medical Research Council (NHMRC).

3. Inclusion and exclusion criteria
Before you decide whether to participate, please know that the study is only recruiting people who:

1. are aged 18 years or older
2. can understand written English
3. have no current moderate to severe mental illness symptoms
4. are an iPhone user residing in Australia, New Zealand, UK, Ireland, USA, or Canada

4. Do I have to take part in this study?
Participation is voluntary. If you do not wish to participate, you can simply use the ReNeuWell® app as a regular user.

5. What does participation require, and are there any risks?
If you decide to participate, you will have 12 weeks of free access to one of two versions of the app. We wish to test which version of the app is more effective for increasing the user's wellbeing, and participants will be randomly assigned to one version or the other. For the first 6 weeks, the app will provide you with daily information and activities, and you will be instructed to use the app for at least 10 minutes a day, 6 days a week. You will take a short online survey before and after this 6-week period. For the final 6 weeks, you will be free to continue using the app as you see fit, and at the end of the 12-week period, you will take the third and final online survey. Each of the three surveys will take about 10 minutes to complete, and will be provided via the app.

The online surveys will ask about your mental health and wellbeing, lifestyle and work habits, and feelings about the app. If you experience any discomfort or distress while participating, you can stop at any time. The contact details of support services will be provided with each survey, and these details are also available below and on the ReNeuWell® NeuRA webpage: https://neura.edu.au/project/reneuwell

6. What are the possible benefits to participation?
Participants will have 12 weeks of free access to the ReNeuWell® app. Using the app may lead to increased mental wellbeing, and the results of this study will be used to develop future programs for improving people's wellbeing.

For peer review only - http://bmjopen.bmj.com/site/about/guidelines.xhtml

7. What will happen to the information I provide?
Your answers to the survey questions will be completely anonymous and confidential. The data will not contain any personally identifying information. In other words, there is no way for the researchers to know the participants' identities. If you decide to participate, the next screen will provide a button labelled "I agree to participate." By clicking this button, you can give your consent to participate.

8. How and when will I find out the results of the study?
The research team will publish the results in scientific journals and other academic outlets. No participant can be identified from these publications. The data will be stored for a minimum of 15 years after publication of the results. A summary of the results will also be available on the ReNeuWell® webpage: https://neura.edu.au/project/reneuwell

9. What if I want to withdraw from the study?
If you decide to participate but then change your mind, you can withdraw at any time, without penalty and without having to give a reason. If you would like to withdraw formally, you will have the option of clicking a button labelled "I wish to withdraw" at each occasion when you are prompted to continue with the study. You can also withdraw via the in-app options menu. After withdrawing, you can uninstall the app or continue using it as a paid user. If you withdraw, you will not be asked to complete any more surveys; however, we will not be able to withdraw your earlier survey answers, because the surveys are anonymous. If you withdraw, you will not be invited to participate in the study again.

10. What should I do if I have further questions about this study?
Please direct any questions about the study to the following research contact:

**Name**	Dr Luke Egan
**Position**	Research Assistant
**Telephone**	02 9399 1883
**Email**	l.egan@neura.edu.au

If you experience any feelings of distress at any stage during the study, or if you require additional support from someone not involved in the research, please access one of the following services (depending on your country of residence):

Australia

**Lifeline**	(telephone support 24 hours a day, 7 days a week)
Telephone:	13 11 14
Web:	https://www.lifeline.org.au/
**Beyond Blue**	(telephone support 24 hours a day, 7 days a week)
Telephone:	1300 224 636
Web:	https://www.beyondblue.org.au/get-support/get-immediate-support

New Zealand

**Lifeline Aotearoa**	(telephone support 24 hours a day, 7 days a week)
Telephone:	0800 54 33 54
Web:	https://www.lifeline.org.nz/

For peer review only - http://bmjopen.bmj.com/site/about/guidelines.xhtml

UK

**Samaritans**	(telephone support 24 hours a day, 7 days a week)
Telephone:	116 123
Web:	https://www.samaritans.org/samaritans-ireland/

Ireland

**Samaritans**	(telephone support 24 hours a day, 7 days a week)
Telephone:	116 123
Web:	https://www.samaritans.org/samaritans-ireland/

USA

**National Suicide Prevention Lifeline**	(telephone support 24 hours a day, 7 days a week)
Telephone:	1800 273 8255
Web:	https://suicidepreventionlifeline.org/

Canada

**Canada Suicide Prevention Service**	(telephone support 24 hours a day, 7 days a week)
Telephone:	1833 456 4566
Web:	https://www.crisisservicescanada.ca/en/

If you have any complaints about any aspect of the study or how it's being run, please contact the UNSW Human Ethics Coordinator and cite the reference number: HC210302.

**Position**	Human Research Ethics Coordinator
**Telephone**	02 9385 6222
**Email**	humanethics@unsw.edu.au

If you have finished reading this Participant Information Statement, please go to the next screen to indicate whether you agree to participate.

Go to next screen →

CONSENT FORM

A randomised controlled trial of the ReNeuWell® wellbeing app

Declaration by the participant:

By clicking the "**I agree to participate**" button below, it will indicate that:

- I understand I am being asked to provide consent to participate in this research study.
- I have read the Participant Information Sheet.
- I provide my consent for the information collected about me to be used for the purpose of this study only.
- I understand that, if necessary, I can ask questions and the research team will respond to my questions.
- I freely agree to participate in the study as described and understand that I am free to withdraw at any time during the study without penalty and without having to give a reason.
- I understand that I can download a copy of the Participation Information Statement and Consent Form from the ReNeuWell® webpage: [insert URL here].

I agree to participate.

I decline to participate.

For peer review only - http://bmjopen.bmj.com/site/about/guidelines.xhtml
